# The role of the C-terminal D0 domain of flagellin in activation of Toll like receptor 5

**DOI:** 10.1371/journal.ppat.1006574

**Published:** 2017-08-21

**Authors:** Vida Forstnerič, Karolina Ivičak-Kocjan, Tjaša Plaper, Roman Jerala, Mojca Benčina

**Affiliations:** 1 Department of Synthetic Biology and Immunology, National Institute of Chemistry, Ljubljana, Slovenia; 2 Centre of Excellence EN-FIST, Ljubljana, Slovenia; University of Illinois, UNITED STATES

## Abstract

Flagellin is a wide-spread bacterial virulence factor sensed by the membrane-bound Toll-like receptor 5 (TLR5) and by the intracellular NAIP5/NLRC4 inflammasome receptor. TLR5 recognizes a conserved region within the D1 domain of flagellin, crucial for the interaction between subunits in the flagellum and for bacterial motility. While it is known that a deletion of the D0 domain of flagellin, which lines the interior of flagella, also completely abrogates activation of TLR5, its functional role remains unknown. Using a protein fusion strategy, we propose a role for the D0 domain in the stabilization of an active dimeric signaling complex of flagellin-TLR5 at a 2:2 stoichiometric ratio. Alanine-scanning mutagenesis of flagellin revealed a previously unidentified region of flagellin, the C-terminal D0 domain, to play a crucial role in TLR5 activation. Interestingly, we show that TLR5 recognizes the same hydrophobic motif of the D0 domain of flagellin as the intracellular NAIP5/NLRC4 inflammasome receptor. Further, we show that residues within the D0 domain play a previously unrecognized role in the evasion of TLR5 recognition by *Helicobacter pylori*. These findings demonstrate that TLR5 is able to simultaneously sense several spatially separated sites of flagellin that are essential for its functionality, hindering bacterial evasion of immune recognition. Our findings significantly contribute to the understanding of the mechanism of TLR5 activation, which plays an important role in host defense against several pathogens, but also in several diseases, such as Crohn’s disease, cystic fibrosis and rheumatoid arthritis.

## Author summary

Receptors of the innate immune system typically recognize conserved microbial patterns, crucial for pathogen fitness and survival. Flagellin, the main structural protein of bacterial flagella, is recognized by two receptors of the innate immune system, the intracellular inflammasome receptor NAIP5/NLRC4 and the membrane-bound Toll-like receptor 5. Ligand-induced dimerization is a crucial step in Toll-like receptor 5 activation. A crystal structure of segments of the ligand-bound receptor revealed binding interfaces on the ligand and receptor, but failed to fully clarify the activation mechanism, since the D0 domain of flagellin, which is crucial for receptor activation, is missing in the structure. We propose a role for the D0 domain in receptor dimerization and pinpoint specific amino-acid residues within the D0 domain, which contribute to Toll-like receptor 5 activation. We show that Toll-like receptor 5 recognizes the same protein motif detected by the intracellular NAIP5/NLRC4 receptor. Our work represents an important advance in the understanding of the mechanism of activation of Toll-like receptor 5.

## Introduction

Toll-like receptors (TLRs) belong to a family of germ-line encoded innate immune receptors able to sense pathogen-associated molecular patterns (PAMPs) [1]. Upon ligand binding, TLRs dimerize, recruiting adaptor molecules that bind to the intracellular TIR domain dimer and induce downstream signaling, resulting in the synthesis of pro-inflammatory cytokines and other immune response effectors [2,3]. Despite a conserved global fold of the structures of TLR receptors, the diversity of ligands they are able to recognize is very broad, ranging from small molecules such as nucleoside analogues to larger molecules such as nucleic acids and proteins. The ability to respond to such a wide array of agonists lies in the distinct recognition mode specific for each TLR and its ligands [4]. Recognition of the characteristic molecular features of microbial ligands (PAMPs) that are essential for the microbial survival and virulence makes it difficult for the pathogen to modify the structure of PAMP in order to circumvent immune recognition without losing its functionality.

Toll-like receptor 5 (TLR5) recognizes flagellin, the main structural protein of bacterial flagella, which exhibits a remarkable level of conservation among bacterial species, thus representing an attractive target for innate immune recognition [5]. Flagellin is composed of multiple structural domains, D0-D3. Flagellin monomers are stacked into a helical filament with the conserved D0 and D1 domains facing inward into the filament core channel, through which flagellin molecules are transported during flagella formation, while the variable domains D2 and D3 protrude outward from the core and are solvent exposed [6]. Functional flagella represent a virulence factor for several important human pathogens [7–9]. Flagellins of β- and γ-proteobacteria, such as *Serratia marcescens* or *Salmonella typhimurium*, are efficiently detected in their monomeric form by TLR5 at pico-molar concentrations, while some bacterial species, such as the Epsilonproteobacteria gastric pathogen *Helicobacter pylori* or the food-borne pathogen *Campylobacter jejuni*, have evolved their flagellin to evade TLR5 recognition while retaining bacterial motility. Several amino acid changes that contribute to TLR5 evasion in *H*. *pylori* FlaA flagellin have been identified within the D1 domain [10]. This evolutionary adaptation involves large restructuring of packing of flagellin monomers into filaments, which comprise 7 molecules of the FlaA of *H*. *pylori* per turn, in comparison to the 11-fold symmetry in flagellin FliC of *S*. *typhimurium* [11].

Mutational and structural studies have identified conserved regions on the TLR5 ectodomain that interact with amino acid residues within the conserved D1 domain of flagellin [12–14]. The crystal structure of the N-terminal fragment of the zebrafish TLR5-N14_VLR_ comprising approximately two thirds of the TLR5 ectodomain in complex with a fragment of *Salmonella* flagellin, lacking the conserved D0 domain, provides a detailed characterization of this interaction. The structure reveals a primary binding interface for α-helices of the D1 domain of flagellin on the ascending lateral surface of TLR5, stretching from leucine-rich repeat LRRNT to LRR10, leading to formation of a 1:1 TLR5-flagellin complex. A secondary but relatively small binding interface mediates the interaction of the αND1b helix and the subsequent β-hairpin region of flagellin with the convex side of LRR12/13 on the opposite TLR5 receptor and is thus proposed to guide the dimerization of the complex in a stoichiometry of 2:2, the physiological relevance of which has also been confirmed in human cells [14,15]. However, the identified interactions do not appear to be sufficient for the formation of an active signaling receptor dimer, since the truncated form of flagellin lacking the D0 domain is insufficient for TLR5 activation [12,16]. Moreover, the 2:2 complex observed in the crystal was not detected in solution, despite binding of flagellin to TLR5 in a 1:1 stoichiometric ratio [14]. A 2:2 complex required for receptor activation was also not observed in a subsequent report on the crystal structure of *B*. *subtilis* flagellin in complex with a fragment of the TLR5 ectodomain [17]. This suggests, in agreement with previous studies, that the truncated fragment of the receptor lacks part of the binding site for flagellin [18]. The D0 domain is essential for functional flagella formation and signaling, yet its deletion only slightly impairs binding to the TLR5 monomer; therefore, the mechanism of its functional role in TLR5 signaling remains unknown [14,16,19].

The motivation to clarify the mechanism of TLR5 signaling and, on the ligand side, to assess the contribution of the D0 region to the evasion of host sensing led us to analyze the role of the D0 domain in receptor activation. We propose a role for the D0 domain in crosslinking the two TLR5 receptor monomers and hence stabilizing the functional signaling complex. While flagellin lacking the D0 domain was incompetent in triggering TLR5 activation, tethering two inactive flagellin D0 deletion variants into a covalent dimer restored activation, suggesting a role for the D0 domain in receptor dimerization. A structure-guided mutagenesis study identified amino acid residues at the C-terminal segment of the D0 domain involved in receptor activation. Further, we pinpointed amino acid residues within the D0 domain that enabled evasion of immune recognition by the *H*. *pylori* flagellin. Together, this shows that the multipartite recognition of flagellin by TLR5 hinders an easy evasion of immune recognition by single point mutations through targeting conserved segments of flagellin, which are essential for flagellar self-assembly.

## Results

### Investigation of the role of the D0 domain using a synthetic biology approach

**Fig 1:**
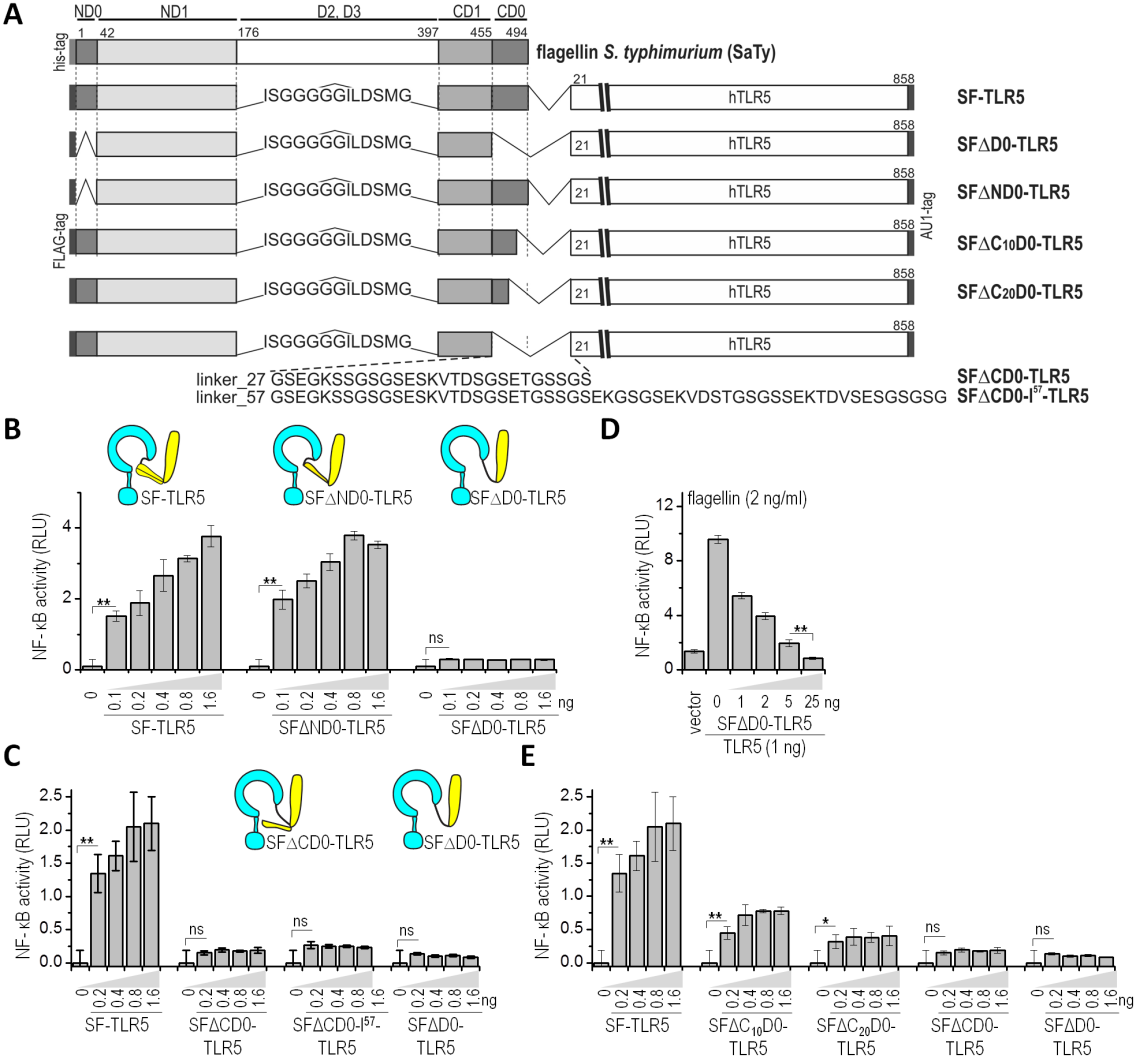
The C terminal D0 domain of flagellin is required for TLR5 activation. **(A)** A schematic representation of the domain organization of S. typhimurium flagellin (SaTy) and of the chimeric constructs comprised of truncated flagellins linked to TLR5. Numbering refers to SaTy (UniProtKB - P06179) and TLR (UniProtKB: O60602). **(B)** Deletion of the N-terminal D0 domain (ND0) does not affect NF-κB activity. HEK293 cells were transfected with increasing amounts (ng) of plasmids encoding ligand-receptor fusions: SF-TLR5 as a positive control, SFΔND0-TLR5, or SFΔD0-TLR5. **(C)** Deletion of the C-terminal D0 domain (CD0) abrogates activity of the constitutively active fusion protein SF-TLR5. HEK293 cells were transfected with increasing amounts (ng) of plasmids expressing ligand-receptor fusions: SF-TLR5, SFΔCD0-TLR5, SFΔCD0-l57-TLR5, or SFΔD0-TLR5 **(D)** The D0 deletion construct SFΔD0-TLR5 forms inactive heterodimers with TLR5. HEK293 cells were transfected with TLR5 (1 ng) and increasing amounts of SFΔD0-TLR5 (1–25 ng). **(E)** Deletion of the C-terminal 10 (SFΔC10D0-TLR5) or 20 (SFΔC20D0-TLR5) amino acid residues of the C-terminal D0 domain decreases activity of the constitutively active fusion protein SF-TLR5. HEK293 cells were transfected with plasmids encoding SF-TLR5, SFΔC10D0-TLR5, SFΔC20D0-TLR5, SFΔCD0-TLR5, or SFΔD0-TLR5. (B-E) HEK293 cells were transfected with plasmids encoding the ligand-receptor fusions and NF-κB-dependent firefly and constitutively expressed Renilla luciferase activities were measured 18 h post-transfection. (Data are representative of three independent experiments. Bars represent the means of four biological replicates ±s.d.; **p<0.005; *p<0.05; nsp>0.05) See also S1 Fig.

To assess the role of the D0 domain of flagellin in TLR5 activation, we used a synthetic biology strategy by constructing a chimeric protein, composed of the D0 and D1 domains of *S*. *typhimurium* flagellin fliC, which we named short flagellin (SF), fused via a flexible peptide linker to the N-terminus of human TLR5 (SF-TLR5). This chimeric protein, combining selected ligand domains and the full-length receptor in a single molecule, exhibited constitutive activity when expressed in HEK293 cells, while a fusion protein lacking the D0 domain (SFΔD0-TLR5) was inactive [15]. Most post-translational modifications of flagellin are located in the variable D2 and D3 domains and it has been shown previously that bacteria-specific post-translational modification of flagellin is not required for TLR5-based recognition [12,20]. The D0 domain is composed of two discontinuous epitopes spanning the N- and C-terminal regions (hereafter referred to as ND0 and CD0), comprising amino acid residues 1–42 and 455–494 of SaTy, respectively (Fig 1A). We aimed to assess the contribution of distinct regions of the D0 domain to TLR5 activation. HEK293 cells were transiently transfected with plasmids for the chimeric proteins and activation of NF-kB reporter was determined through a dual luciferase reporter assay (Fig 1). SF-TLR5 was used as a positive and SFΔD0-TLR5 as a negative control. A fusion protein variant comprising short flagellin lacking the ND0 domain (SFΔND0-TLR5) retained the constitutive activity of the positive control, SF-TLR5 (Fig 1B). On the other hand, deletion of the C-terminal D0 domain (SFΔCD0-TLR5) completely abrogated NF-κB activation, as did the negative control, a chimeric receptor lacking the complete D0 domain (SFΔD0-TLR5) (Fig 1C). One explanation for this effect might be that the truncation of the CD0 domain in the chimeric construct shortens the distance between flagellin and TLR5 and could thus lead to a steric hindrance in binding. To rule out this possibility and confirm that the truncation of the C-terminal flagellin segment on its own is the cause of the impaired signaling, we tested constructs with two longer linker lengths, 27 and 57 aa (Fig 1A), which resulted in the same level of activation (Fig 1C). To rule out inactivation of the truncated constructs due to protein misfolding, a cotransfection assay with wtTLR5 was performed. The results showed a decrease in wild-type TLR5 activation by flagellin upon addition of increasing levels of the inactive SFΔD0-TLR5, suggesting an interaction between wt-TLR5 and TLR5-SFΔD0, which is inactive but can still bind TLR5 and can therefore inhibit TLR5 activation (Fig 1D). Western blot analysis confirmed that the expression level of wtTLR5 remains constant, even upon expression of increasing levels of the fusion receptor SFΔD0-TLR5 (S1B Fig). The decrease in signaling is therefore not due to a limited cell capacity for overexpression of recombinant proteins.

Further, we prepared deletion constructs lacking the C-terminal 10 (SFΔC_10_D0-TLR5) or 20 (SFΔC_20_D0-TLR5) amino acids of the CD0 domain (Fig 1A). The NF-κB luciferase reporter assay showed a decrease in signaling proportional to the size of the deletion, although complete abrogation of signaling was observed only upon deletion of the complete C-terminal D0 domain (Fig 1E). All proteins where detected in cell lysates at comparable levels, confirming that the decrease in activation is not a consequence of altered protein expression (S1A Fig). These results underline a prominent role for the conserved C-terminal D0 domain of flagellin in TLR5 activation.

### Identification of residues in the C-terminal D0 domain of flagellin that affect TLR5 signaling

**Fig 2:**
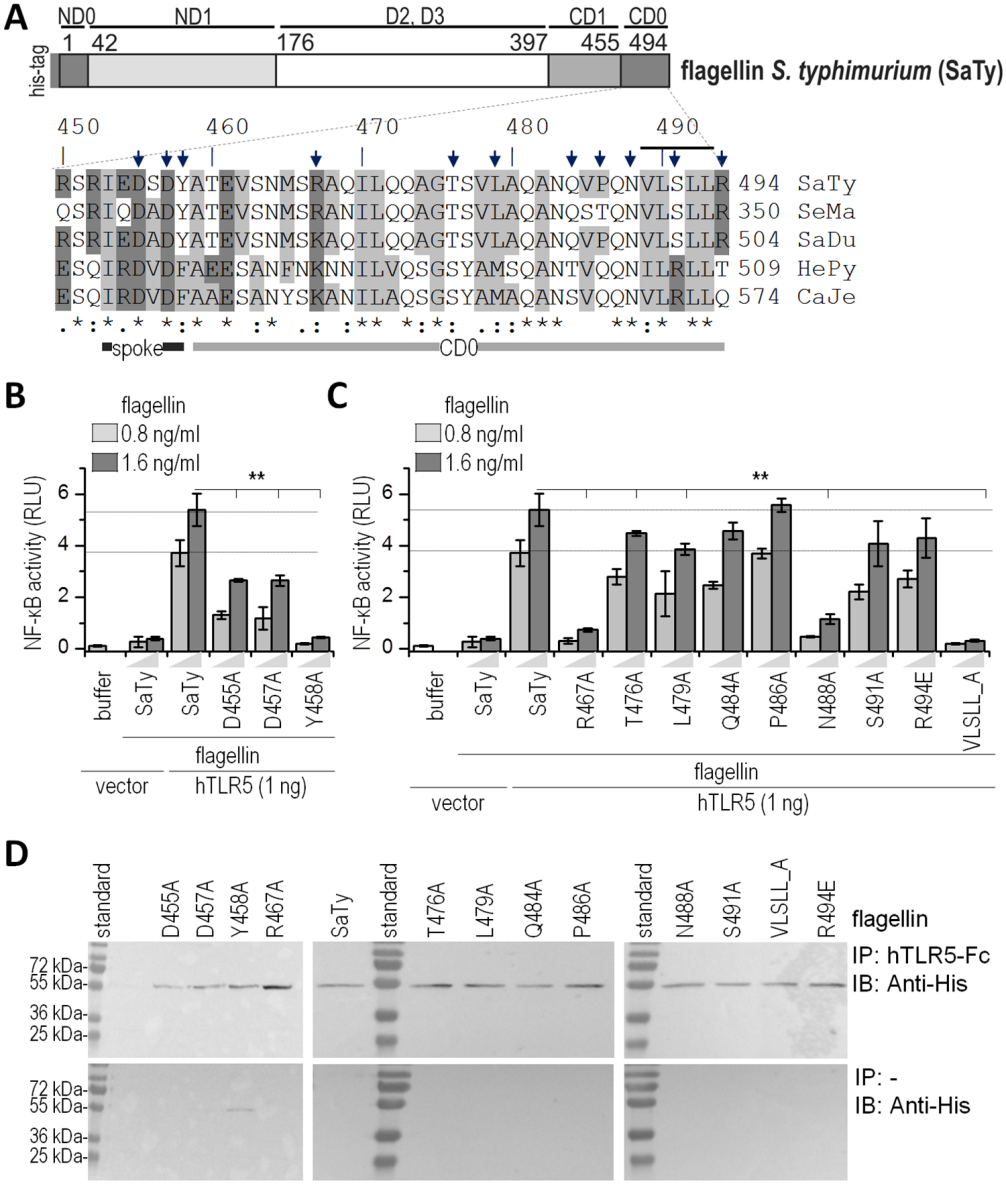
Conserved amino acid residues at the C-terminus of flagellin affect TLR5 activation but not the binding of flagellin to TLR5. (A) Amino acid alignment of C-terminal flagellin sequences of several bacterial species and amino acid residues selected for alanine scanning mutagenesis (indicated with arrows) and sequence VLSLL (indicated with line). SaTy–*S*. *typhimurium*; SeMa–*S*. *marcescens*; SaDu–*S*. *dublin*; HePy–*H*. *pylori*; CaJe–*C*. *jejuni*. Numbering refers to *S*. *typhimurium* flagellin. (B, C) Activation of TLR5 stimulated with recombinant wild type flagellin, C-terminal spoke (B) and D0 mutants (C). HEK293 cells were transfected with a plasmid encoding hTLR5 (1 ng) or an empty vector (as negative control) and stimulated with SaTy flagellin or mutants (0.8 or 1.6 ng/ml). After 18 h NF-κB-dependent firefly and Renilla luciferase activities were measured (Data are representative of three independent experiments. Bars represent the means of four biological replicates ±s.d.;**p<0.005). (D) Co-immunoprecipitation was performed to assess the binding of the mutated proteins to human TLR5. 20 μg of each protein was added to protein-A conjugated beads with bound hTLR5-Fc (top) or control beads (bottom). Anti-His antibodies were used for the detection of wild-type flagellin and mutants. Data are representative of two independent experiments. See also S2 Fig.

Based on the alignment of flagellins of different bacterial species, amino acid residues were selected for alanine mutagenesis. The selection criteria included amino acid residues of the *Salmonella* flagellin FliC, which differ from their *H*. *pylori* FlaA counterparts, but also residues that are conserved among the different clades (Fig 2A). Alanine point mutations were introduced, with the exception of a substitution of the terminal arginine R494 to glutamic acid since an alanine mutant at this site was highly prone to proteolysis in bacterial overexpression. In addition to single alanine point mutations in the CD0 domain, a substitution of the hydrophobic motif in the region 489 to 493 (VLSLL) by five alanine residues was introduced (VLSLL_A) (Fig 2A). Recombinant flagellins were produced and isolated via affinity chromatography (S2A Fig) and tested for TLR5 activation potential in two reporter cell lines: HEK293 cells transiently expressing human TLR5 (Fig 2B and 2C) and the human epithelial cell line A549, which endogenously expresses TLR5 (S2B–S2E Fig). Mutations D457A and Y458A in the conserved spoke region showed a strong negative effect on TLR5 activation. A mutation in the central region of CD0, R467A, had a profound effect on activation, while other mutations in the central region had a low or no apparent effect on the activation of the NF-κB signaling pathway. Substitution of the hydrophobic amino acid residues at the very tip of the protein VLSLL_A completely abrogated TLR5 signaling, while a single point mutation of serine (S491A) within this motif had no effect, thus attributing the effect of protein VLSLL_A entirely to the hydrophobic residues. Mutation of the polar residue N488A, located at the tip of the CD0 domain preceding the hydrophobic motif VLSLL also significantly impaired signaling. Additionally, regions 460–463 (TEVS) and 472–474 (QQA) within the CD0 domain, which differ between *S*. *typhimurium* and *H*. *pylori* flagellin (SaTy and HePy, respectively), were mutated from SaTy to the corresponding residues of HePy (TEVS_EESA and QQA_VGS). The isolated recombinant proteins were tested for TLR5 activation (S2F Fig). However, these substitutions showed no effect on TLR5 activation, suggesting that the residues in the spoke region and at the C-terminus are crucial for TLR5 activation.

The mutant protein VLSLL_A was also tested for activation of the intracellular NAIP5/NLRC4 inflammasome. Wild type or NLRP3-deficient macrophages were primed with LPS and stimulated with wt or mutant flagellin and IL-1β secretion was measured. Mutation of the terminal hydrophobic residues resulted in decreased NAIP5/NLRC4 inflammasome activation, in agreement with a previous report (S2H and S2I Fig) [21].

Co-immunoprecipitation studies showed a comparable binding intensity of recombinant mutated flagellins to the TLR5 ectodomain, regardless of their activation potential, which is in agreement with previous studies where a deletion of the D0 domain completely abrogated signaling but hardly affected the binding efficiency of a truncated flagellin molecule to TLR5 [14] (Fig 2D).

These results suggest that the role of the D0 domain in TLR5 activation is largely defined by amino acid residues in the conserved C-terminal spoke region and in the C-terminal hydrophobic tip of flagellin. However, these point mutations do not significantly affect the binding of flagellin to hTLR5, as the primary binding site is located within the D1 region, as shown by previous structural studies [14].

### Mutations in the C-terminal region of flagellin impair bacterial motility

**Fig 3:**
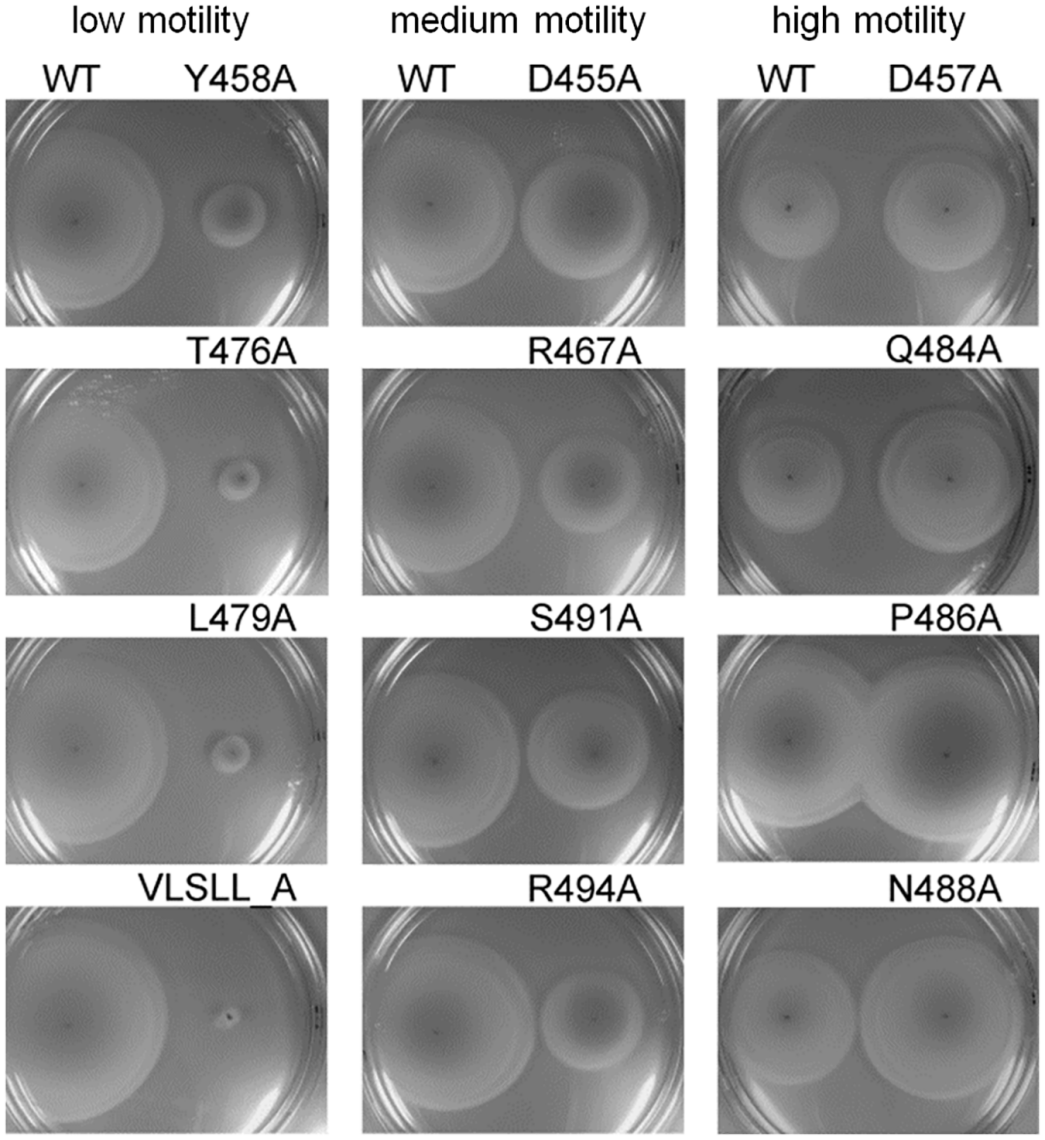
Amino acid residues in the C-terminal region of flagellin are important for bacterial motility. A flagellin deficient strain of *S*. *typhimurium* was transformed with plasmids encoding wt or mutant flagellin and inoculated on soft agar plates to assess the effect on bacterial motility. Each agar plate contains one tested mutant (right) and the wild-type control (left). Mutations are grouped into three categories with respect to their effect on motility. Data are representative of three independent experiments.

In innate immune recognition, a correlation is often observed between function of the PAMP or its structural moiety for the microbe and recognition by TLRs, as structural or functional restrictions often hinder modifications of PAMPs that would enable immune evasion. Previous reports suggested a correlation between TLR5 activation and effects on the motility in the conserved D1 region of flagellin [10,22]. Amino acids in the C-terminal region of flagellin are also evolutionarily conserved among bacterial species since they tile the inner channel of the flagellum and participate in packing of neighboring flagellin chains. To assess whether there is a correlation between TLR5 recognition and motility for this region of flagellin, we tested mutated flagellins for their effect on functional flagella formation using a swarming motility assay (Fig 3). The effect on bacterial motility could be grouped into three categories: mutations exerting low motility (left panel), mutations with a moderate effect on motility (middle panel), and mutations which increased bacterial motility with respect to wild-type flagellin (right panel). Among the mutations with a significant effect on TLR5 activation, Y458A in the spoke region and VLSLL_A at the C-terminus had the most profound effect on motility while mutation R467A had a moderate effect. These results show that, while the majority of mutations that impair TLR5 activation also have a negative effect on motility, there is no direct correlation between TLR5 stimulation and function, at least not at the amino acid level.

### The functional role of the D0 domain of flagellin in stabilizing the dimeric receptor complex

**Fig 4:**
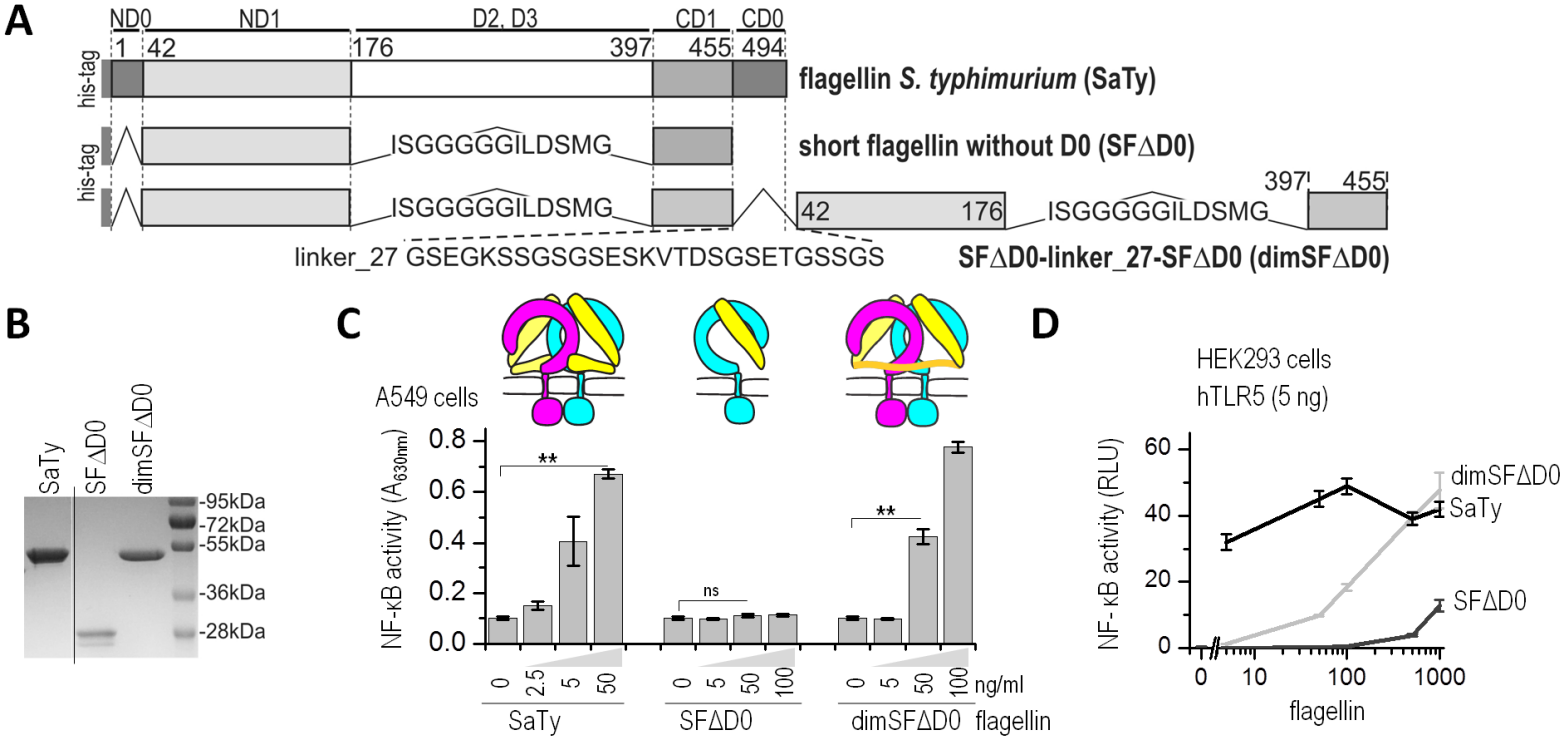
The D0 domain of flagellin mediates TLR5 activation through crosslinking. (A) A schematic representation of the domain organization of SaTy, SFΔD0, and a dimer of SFΔD0 tethered by peptide linkers. Numbering refers to *S*. *typhimurium* flagellin. (B) SDS-PAGE of purified recombinant proteins. (C) SFΔD0 is unable to activate TLR5 signaling, whereas activation with a tethered variant dimSFΔD0 has improved stimulation potential. (Above) Schematic representation of the role of the D0 domain in TLR5 activation. SFΔD0 (yellow) binds to but fails to activate TLR5 (cyan, magenta), whereas the linker in a dimer of SFΔD0 mimics the role of the D0 domain in crosslinking two ectodomains, therefore inducing active dimer formation. The human lung epithelial A549 cells were stimulated with SaTy flagellin, SFΔD0, or dimSFΔD0, and NF-κB-dependent SEAP activities were measured. (D) HEK293 cells transfected with a plasmid encoding hTLR5 were stimulated with increasing concentrations (0–1000 ng/ml) of recombinant proteins (SaTy, SFΔD0, or dimSFΔD0) and the NF-κB-dependent firefly and Renilla luciferase activities were measured 18 h later. (Data are representative of three (C) or two (D) independent experiments. Bars or points represent the means of 4 biological replicates ±s.d.; **p<0.005, nsp>0.05,) See also S4 Fig.

For the formation of an active signaling complex, two flagellin molecules must bind to two TLR5 ectodomains [15], forming an active complex in which a dimer of the intracellular TIR domains initiates recruitment of the signaling adapter MyD88. Flagellin binds to TLR5 via a two-partite primary binding site and to the opposing TLR5 through a secondary site, both encompassed in the D1 domain of flagellin [14]. Superposition of full-length flagellin from the assembled flagellum (PDB code 1UCU) to the truncated FliC-ΔD0 from the crystal structure TLR5-N14_VLR_/FliC-ΔD0 demonstrates that in the extended form, the D0 domain of flagellin would clash with the cell membrane (S3 Fig). We reasoned that, upon binding of flagellin to TLR5, the D0 domain must reorient itself relative to the conformation in the assembled flagella. Taking into account this information and the necessity of TLR5 ectodomain dimerization for activation but not for flagellin binding, we hypothesized that the D0 domain might have a role in dimer formation by binding to the opposite TLR5 ectodomains and stabilizing an active complex by bringing the two 1:1 complexes of flagellin:TLR5 into sufficient proximity for signal transduction mediation by the dimerized cytosolic TIR domains. To test this hypothesis, we prepared a recombinant truncated flagellin molecule lacking the D0 and variable D2 and D3 domains (SFΔD0) and a recombinant protein in which the two SFΔD0 domains are tethered into a single polypeptide chain by a 27 amino acid peptide linker (dimSFΔD0) (Fig 4A). SDS PAGE (Fig 4B) and particle size analysis determined by dynamic light scattering (DLS) confirmed the expected size of dimSFΔD0 (6.0 nm) as being approximately twice the size of the monomeric SFΔD0 (2.3–2.5 nm) and comparable to the size of the full-length SaTy flagellin (6.1 nm). The monomeric SFΔD0 was unable to stimulate TLR5 (Fig 4C and 4D A549 cells, S4A and S4B Fig hTLR5-transfected HEK293 cells) in agreement with previous findings [14]. If the role of D0 is indeed to crosslink the two TLR5 ectodomains into a functional signaling dimer, tethering of the truncated flagellin fragment should improve activation. Indeed, in contrast to SFΔD0, dimSFΔD0 was active at concentrations as low as 50 ng/ml (Fig 4A and 4D). Tethering the two inactive short flagellins therefore substantially restores activation, albeit not to the full extent of wild type flagellin. We propose that the linker to a certain extent substitutes the protein-protein interactions mediated by the D0 domain in the full-length flagellin-TLR5 heterodimeric complex. Together, our results suggest a role for the D0 domain in crosslinking two 1:1 TLR5:flagellin complexes into an active 2:2 signaling complex.

### Contribution of residues in the CD0 domain of *H*. *pylori* FlaA to the evasion of TLR5 activation

**Fig 5:**
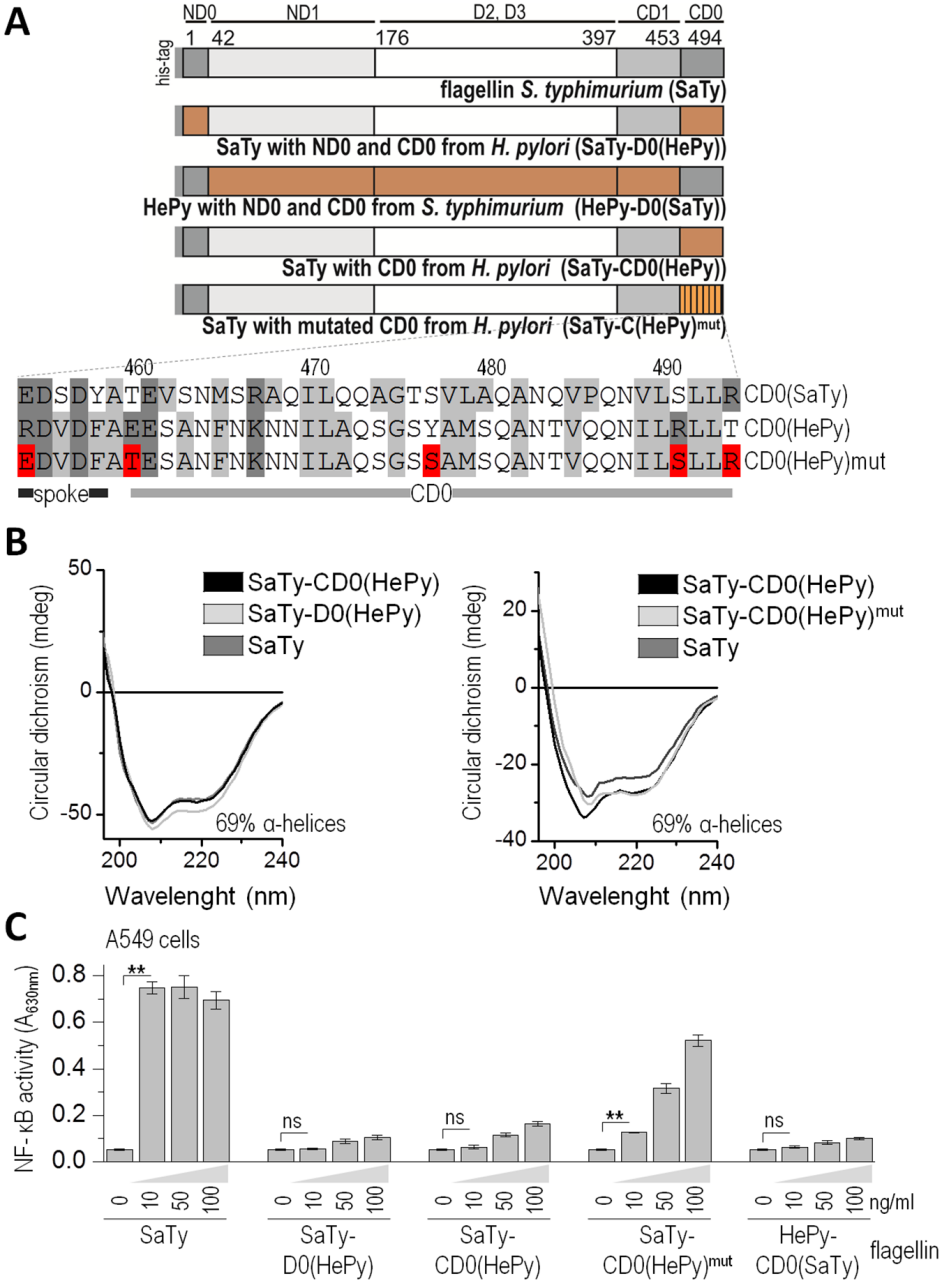
The D0 domain of *H*. *pylori* flagellin contributes to the evasion of TLR5 recognition. (A) Schematic representation of recombinant flagellins composed of SaTy and HePy. The terminal 41 amino acid residues of SaTy where exchanged with those of HePy flagellin to assess the contribution of the CD0 domain to receptor evasion. A selection of less conserved amino acid residues within the HePy CD0 domain was mutated back to the SaTy counterparts to pinpoint the specific modifications allowing this evasion (SaTy-CD0(HePy)mut). (B) Circular dichroism measurements of SaTy and chimeric flagellins confirm a similar fold and exclude the possibility of deleterious effects of protein modifications on structure. Data are representative of two independent experiments. (C) Mutations of specific selected amino acid residues within the CD0 domain partially restore receptor activation. The human lung A549 cells were stimulated with recombinant SaTy flagellin or chimeric proteins. NF-κB-dependent SEAP activities were measured. (Data are representative of two independent experiments. Bars represent the means of 4 biological replicates ±s.d.; **p<0.005, nsp>0.05) See also S5 Fig.

Despite a high level of sequence conservation in the D1 and D0 domains of flagellin, the quaternary filament structures differ between the two proteobacteria clades [6,11]. *H*. *pylori* belongs to the clade of ε-proteobacteria that form a distinct flagellar filament assembly composed of 7 rather than of 11 protofilaments in the flagellum and are able to evade immune recognition by TLR5. A study by Andersen-Nissen et al. (2005) identified mutations in the primary binding site located within the D1 domain that could contribute to the evasion of immune recognition at the cost of impaired motility and compensatory mutations, which restored functional flagella formation and mobility. However, the D0 domain of flagellin also plays a crucial role in flagellar filament assembly by forming contacts with other monomers within the inner channel. To assess the role of the D0 region in the evasion of TLR5 detection by *H*. *pylori*, chimeric flagellins were constructed by exchanging the D0 domain of SaTy with the C-terminal D0 domain of HePy (SaTy-CD0(HePy)) or both the C- and N-terminal D0 domains of HePy (SaTy-D0(HePy)) (Fig 5A). All chimeric flagellin variants were produced in a bacterial expression system and purified via affinity chromatography (S5A Fig). These chimeric proteins comprised both the primary and secondary binding sites of SaTy. Therefore, any difference in the ability of these variants to activate TLR5 depends on the differences between the SaTy and HePy D0 domains. Circular dichroism analysis of chimeric flagellins demonstrated comparable secondary structure content to that of the wild type flagellin, indicating no deleterious effects on protein folding (Fig 5B). Both chimeric flagellins, SaTy-D0(HePy) and SaTy-CD0(HePy) were unable to activate TLR5 either at the endogenous level or ectopic TLR5 expression (Fig 5C human lung epithelial cell line A549, S5B Fig hTLR5-transfected HEK293 cells), similar to a protein completely lacking the D0 domain (SFΔD0), demonstrating the crucial role of the CD0 domain of flagellin in TLR5 activation. Chimeric flagellin of *H*. *pylori* with the C- and N-terminal D0 domains of SaTy also failed to activate TLR5 (Fig 5C, S5C Fig). The chimeric proteins SaTy-CD0(HePy) and SaTy-D0(HePy) had a similar secondary structure to SaTy, as demonstrated by the analysis of the circular dichroism spectra of the isolated proteins. This indicates that the evolutionary adaptations that allowed for the immune evasion of *H*. *pylori* are distributed across the whole length of flagellin and are not restricted to the primary TLR5 recognition surface located within the D1 region.

The amino acid sequences of HePy and SaTy flagellin are highly conserved in the D0 region, pointing to a high level of evolutionary constraints in this area. However, several residues represent more pronounced differences in charge or hydrophobicity. Therefore, we tested a combination of five substitutions from HePy to SaTy counterparts to identify the role of these residues in the evasion of TLR5 activation (Fig 5A). Isolated chimeric protein SaTy-CD0(HePy)^mut^ with the selected counterpart mutations showed a significant recovery of the TLR5 activation potential in comparison to the chimeric protein SaTy-CD0(HePy), suggesting that these particular amino acid differences contribute to immune evasion by *H*. *pylori* (Fig 5C, S5D Fig).

## Discussion

A crucial step in the detection of PAMPS and active complex formation of TLRs is receptor ectodomain dimerization. Despite the important insight into the mechanism of ligand binding by TLR5 from the crystal structure of the complex of the ligand and receptor fragments, important aspects of TLR5 activation by flagellin remain unknown. A distributed binding site on the concave and lateral surfaces of TLR5, extending from LRRNT to LRR10, directs the primary binding of flagellin, enabling formation of a TLR5:flagellin 1:1 complex. A secondary binding site between the D1 domain of flagellin and LRR12-13 of the opposing TLR5 contributes to the formation of a 2:2 complex. These observed interactions are however not sufficient for receptor activation *in vivo*, as the truncated flagellin lacking the D0 domain is not able to trigger signaling. Furthermore, a subsequent study reported weak binding of flagellin to the ectodomain region beyond LRR17, not included in the crystal structure [23]. The unresolved issues concerning the TLR5 activation mechanism motivated us to investigate the role of the D0 domain in TLR5 activation in greater detail.

Results of a flagellin subdomain deletion revealed a key role of the C-terminal segment of the D0 domain in TLR5 activation. Further, a detailed alanine-scanning mutagenesis of this region revealed the contribution of several amino acid residues to TLR5 activation. Substitutions of two amino acid residues in the spoke region significantly affected receptor activation. The most conserved residues across all flagellins are those in the spoke region. The spoke region is crucial in filament formation for maintaining the integrity of the inner and outer tubes of the filament, and it forms inter-subunit interactions that enable tight packing of flagellin monomers [5]. A pronounced role in TLR5 activation was also demonstrated for amino acids at the very tip of flagellin, including polar asparagine at position 488 and the hydrophobic motif VLSLL from positions 489 to 493, excluding Ser491. While this region has not been previously reported to have a role in TLR5 activation, the same terminal hydrophobic motif is also crucial for recognition by the intracellular NAIP5/NLRC4 inflammasome [21]. Our results therefore suggest a dual mechanism of sensing the same region of bacterial flagellin through two distinct receptors of innate immunity. Although the molecular mechanism of flagellin recognition by NAIP5 is not known and is likely to differ from the mechanism of recognition by TLR5, selection of the same segment as the target for the innate immune receptors is likely due to the functional importance of this region for the assembly of functional flagella.

In the crystal structure study, Yoon et al. demonstrated that the contribution of the D0 domain to the formation of the 1:1 complex of flagellin:TLR5 is minimal [14]. Therefore, unless there is a third unknown co-factor involved in the TLR5-flagellin signaling event, the contribution of the D0 domain is most likely pertained to the formation of the 2:2 complex, required for activation. In line with these findings, we showed that, while point mutations within the C-terminal D0 domain strongly decreased signaling, binding of flagellin to TLR5 was not affected. Superposition of full-length flagellin to the TLR5 dimer based on the crystal structure TLR5-N14_VLR_/FliC-ΔD0 demonstrates that the structure of TLR5-bound flagellin must differ from the structure of flagellin in filaments. The spoke region connecting D0 and D1 is highly conserved among bacterial species and represents a flexible subunit of the flagellin molecule between domains D0 and D1, which are mostly α-helical in flagellar filaments, whereas the D0 domain is disordered in monomeric flagellin [6,24]. We propose that the flexible spoke region connecting the D0 and D1 domains functions as a hinge, enabling the reorientation of the D0 domain toward the opposing TLR5 ectodomain and thus stabilizing the 2:2 complex. This is supported by the recovered activity of a completely inactive form of flagellin without the D0 domain (SFΔD0) by forced dimerization in a covalently tethered SFΔD0 dimer. One might argue that increased activation of the dimeric construct could be simply due to increased local concentration of the ligand, although we believe this to be less likely, since the monomeric form of flagellin lacking the D0 domain is essentially inactive, even in a higher concentration range. Our results therefore identify a previously unrecognized segment of flagellin to play an essential role in TLR5 activation and suggest a role for the D0 domain in the process of receptor dimerization most likely through binding to the opposing ectodomain in the active complex. We note that these results provide indirect evidence to the mechanism of TLR5 dimerization and that direct proof of this concept would have to be supported by a structural study of the full length form of the ligand-receptor complex. However, structural studies of the D0 domain in the monomeric form of flagellin have, at least do date, been unsuccessful, most likely due to the disordered structural conformation of the terminal regions of flagellin [6,14,17,24].

Inter- and intra-subunit interactions of the D1 and D0 domains enable the tight packing of flagellin subunits into functional filaments [6]. The high level of sequence conservation in these regions illustrates their functional importance, and single point mutations can disrupt the proper quaternary structure and therefore bacterial motility. Indeed, bacterial motility was significantly reduced for several flagellin mutants. Inter-subunit connections between the D0 domain in the filament core are mostly hydrophobic [6,25], and therefore it is not surprising that of the mutants that affected TLR5 activation, the two hydrophobic mutations, Y458A and VLSLL_A, located in the spoke region and at the C-terminal tip, profoundly impaired motility, exhibiting a link between structure and function as an ideal target for immune recognition. These residues are directly involved in packing interactions with neighboring flagellin molecules in the filament. Main-chain and side-chain atoms from residues at the C-terminus of flagellin, including Q484, N488, S491, and R494, constitute the hydrophilic surface of the inner channel, which is important for the transport of monomeric flagellin through the channel in the process of filament formation [6,26]. While mutations at positions S491 and R494 weakly impaired motility, mutations at positions N488 and Q484 even increased motility with respect to wt flagellin. Retained and even increased bacterial motility might be explained by the position of residues N488 and Q484, which are oriented towards the center of the hydrophilic channel, where they do not participate in filament packing. Mutation of asparagine into alanine is expected to increase the size of the channel, without significantly affecting its polarity due to the small size of the alanine side chain, presumably facilitating transport as a result of increased channel size.

The distribution of the TLR5 binding sites across the length of the flagellin molecule includes several conserved segments of flagellin within the D1 and D0 domains required for flagellin self-assembly [12], thereby rendering the evasion of immune recognition rather difficult, requiring multiple coordinated mutations. The extent of the required change is illustrated by radically different packing of flagellar filaments in ε-proteobacteria, such as *H*. *pylori*. In addition to the previously recognized contribution of the D1 domain, we demonstrated a role of the D0 domain in the evasion of TLR5 recognition based on the exchange of the D0 domain of a potent TLR5 activator, *S*. *typhimurium* flagellin (FliC), with the D0 domain of the *H*. *pylori* flagellin (FlaA), which is unable to trigger TLR5-based immune activation. Further, specific amino acid residues have been identified, which had to be altered in the evolution of *H*. *pylori* to enable this evasion. We may speculate that the complex rearrangement of flagellin sequences that modify the supramolecular structure of flagella and evade TLR5 recognition may be easier in *H*. *pylori* due to its ability to recombine the genetic material from several strains within the same organism and therefore simultaneously combine multiple point mutations within the same molecule [27].

In conclusion, we suggest a functional role of the D0 domain of flagellin in TLR5 activation through the promotion of receptor dimerization. We propose a mechanism for the formation of a fully active TLR5:flagellin complex, in which the contribution of at least three distinct sites on flagellin is required; the primary binding site within the D1 domain that guides the formation of the flagellin:TLR5 heterodimer, and the secondary binding site that promotes interaction between flagellin and the opposite TLR5 ectodomain [14], which needs to be supported by an additional third interaction between the D0 domain of flagellin and the opposing TLR5 ectodomain. The multiple interaction surfaces identified in this and previous studies [12–14] contribute to the high affinity of binding and underlay the evolutionary robustness of TLR5 recognition. The importance of the D0 domain in the self-assembly of flagella makes it an excellent choice for a recognition target and it is not surprising that two completely different types of receptors of the innate immune system target the same region of the molecule. In fact, the membrane TLR4 and cytosolic caspase 11 receptors also recognize a very similar structural pattern of the LPS molecule [28], demonstrating the convergent evolution of the innate immune system to the functionally most relevant microbial targets.

## Materials and methods

### Cell cultures and plasmids

Human embryonic kidney cell lines HEK293 (ATCC CRL-157) and HEK293T (ATCC CRL-3216) and A549-Dual adherent epithelial cells (Invivogen) were cultured in complete media (DMEM; 1 g/l glucose, 2 mM L-glutamine, 10% heat-inactivated FBS (Gibco)) in 5% CO_2_ at 37°C. The human A549 lung carcinoma cell line expresses a secreted embryonic alkaline phosphatase (SEAP) reporter under the control of the IFN-β minimal promoter fused to five NF-κB binding sites, and a secreted luciferase under the control of an ISG54 minimal promoter in conjunction with five IFN-stimulated response elements (Invivogen). The plasmids used include pUNO-hTLR5 encoding human TLR5 (InvivoGen) and pcDNA3 (Invitrogen). *S*. *typhimurium* flagellin, chimeric flagellins, and flagellin point mutants were cloned into the pET19b expression vector (Novagen). For the bacterial motility assay, flagellin mutants were cloned into the pRP4 plasmid expressing wild-type flagellin (courtesy of E. Miao, Institute for Systems Biology, Seattle). Further, chimeric proteins SFΔD0, dimSFΔD0, and chimeras of SaTy and HePy flagellin were cloned into the pET19b expression vector (Novagen). Fusions of short flagellins with TLR5, SF-TLR5, SFΔD0-TLR5 [15], SFΔCD0-TLR5, SFΔCD0-l^57^-TLR5, SFΔND0-TLR5, SFΔC_10_D0-TLR5, and SFΔC_20_D0-TLR5 were cloned into the pFLAG-CMV3 expression vector (Sigma-Aldrich). The chimeric constructs prepared for this study are described in detail in S1 Table.

### Production and isolation of bacterial flagellins

*Escherichia coli* BL21 (DE3) pLysS cells transformed with the pET19b plasmid expressing wild-type or mutant flagellin were cultivated at 37°C in Luria-Bertani (LB) medium, containing 50 μg/ml ampicillin. Overnight cultures were transferred to fresh media, grown to an optical density of ~0.8 at 600 nm, and supplemented with 1 mM Isopropyl β-D-thiogalactoside (IPTG). Cells were grown at 37°C for 4 hours, harvested, and lysed in buffer (10 mM TRIS pH 7.5, 1 mM EDTA, 0.1% DOC) containing a protease inhibitor cocktail (Sigma P8849), followed by sonication (pulse 1 s on, 2 s off, 10–15 min) and centrifugation (12000 rpm for 30 min). N-terminally His_10_-tagged recombinant proteins were purified on Ni-NTA affinity agarose (Qiagen) and dialyzed against 20 mM HEPES buffer. Flagellin point mutants prone to protease degradation (D455A, D457A, Y458A, and T476A) were additionally purified via a Strep-tag on the C-terminus on a Strep-Tactin Sepharose column (Iba), according to the manufacturer’s guidelines. Protein concentration was determined with the BCA assay (Pierce), and purity was confirmed using SDS-PAGE and immunoblotting.

### Luciferase reporter assay

For the dual-luciferase assays, HEK293 cells were seeded in 96-well plates (Corning) at 2–3 × 10^4^ cells per well (0.1 ml). The next day, the cells were transiently transfected with plasmids expressing wtTLR5 or chimeric constructs, pELAM-1 (C. Kirschning, University of Duisburg-Essen, Germany) expressing NF-κB-dependent firefly luciferase (50 ng per well), and phRL-TK (Promega) constitutively expressing Renilla luciferase (5 ng per well) using the jetPEI transfection reagent (Polyplus Transfection). The total amount of DNA for each transfection was kept constant by adding appropriate amounts of control plasmid pcDNA3 (Invitrogen). After 24 h, the cells were either lysed or the medium was changed and the cells were stimulated with purified recombinant flagellin (10 μl) for an additional 18 h before lysis. The cells were lysed in Passive Lysis 5x Buffer (Promega) and analyzed for reporter gene activities using a dual-luciferase reporter assay. Each experiment was repeated at least three times, and each measurement was performed in at least four biological parallels. A student’s unpaired two-tailed t-test was used for statistical comparison.

### SEAP reporter assay

A549 epithelial cells express endogenous TLR5 and an NF-κB-inducible SEAP reporter and do not express the intercellular reporter for flagellin, NLRC4 [29]. Cells were seeded in 96-well plates (Corning) at a density of 5 × 10^4^ cells per well (0.1 ml). Immediately after seeding, the A549 cells were stimulated with flagellin. After 10 h, the supernatants were collected, heated for 1 h at 65°C, and the NF-κB-dependent SEAP activity was determined using Quanti Blue reagent according to the manufacturer’s instructions (Invivogen). Each experiment was repeated at least three times, and each measurement was performed in at least five biological parallels. A student’s unpaired two-tailed t-test was used for statistical comparison.

### Immunoblotting

HEK293T cells were seeded in a 6-well plate (Techno Plastic Products) at a density of 5–7 × 10^5^ cells per well. The next day, the cells were transiently transfected with 2 μg of plasmids expressing TLR5 or TLR5 fusion constructs using the Lipofectamine transfection reagent (Thermo Fisher Scientific). In the case of transfection of varied amounts of plasmids, the overall amount of transfected DNA was equalized using control plasmid pcDNA3. Forty-eight h post transfection, the cells were lysed in lysis buffer (50 mM Tris-HCl (pH 8), 1 mM EDTA, 1 mM EGTA, 137 mM NaCl, 1% Triton X-100, 1% sodium deoxycholate (DOC), 10% glycerol, 1 mM Na_3_VO_4_, and 50 mM NaF) containing a cocktail of protease inhibitors (Roche). Cell debris was removed by centrifugation at 13200 rpm for 15 min. The total protein concentration in the supernatant was determined using the BCA assay. Further, proteins from the supernatant were separated by SDS-PAGE and transferred to a Hybond ECL nitrocellulose membrane (GE Healthcare). The membrane was washed (1 × PBS buffer) and incubated in blocking buffer (1 × PBS, 0.1% Tween 20, 0.2% I-Block (Tropix)) overnight at 4°C. The membranes were incubated with primary antibodies diluted in blocking buffer for 90 min, washed (1 × PBS, 0.1% Tween 20), and incubated with secondary antibodies for 45 min at room temperature. Secondary antibodies were detected with the ECL Western blotting detection reagent (GE Healthcare), according to the manufacturer’s protocol. The primary antibodies were rabbit anti-FLAG (F7425, Sigma), rabbit anti-AU1 (ab3401, Abcam;) and mouse β-aktin (Cell Signaling techn., 3700;), all diluted 1:1000. Secondary antibodies were horseradish peroxidase-conjugated goat anti-rabbit IgG (ab6721, Abcam) and HRP-conjugated goat anti-mouse IgG (Santa Cruz, sc-2005), both diluted 1:4000. Further, the purity of the recombinant flagellins isolated from *E*. *coli* was analyzed by SDS-PAGE and western blot analysis using mouse Tetra-His antibodies diluted 1:2000 (34670, Qiagen) and horseradish peroxidase-conjugated goat anti-mouse IgG diluted 1:4000 (sc-2005, Santa Cruz) as secondary antibodies.

### Co-immunoprecipitation binding assay

For the co-immunoprecipitation studies, a soluble hTLR5 ectodomain fused to the Fc region of human IgG1 (Invivogen) was bound to protein A-coupled Dynabeads (Thermo Fisher Scientific), according to the manufacturer’s protocol. A total of 10 μg of hTLR5-Fc diluted in MQ water or just MQ water as a control was incubated with 40 μl of beads for 1 h at room temperature and washed 3 times with wash buffer (1 × PBS, 0.02% Tween 20, pH 7.5). Twenty μg of wt or mutant flagellin was added per sample, incubated for 1 h at room temperature, and washed 3 times with wash buffer. The samples were eluted in 0.1% SDS at 95°C for 5 min. The flagellin in the samples was detected with Western blot analysis using anti-His antibodies.

### Bacterial motility assays

The impact of flagellin mutations on bacterial motility was tested using an immobile bacterial strain, *S*. *typhimurium Fli*C *Flj*B ATCC 14028s (courtesy of E. Miao, Institute for Systems Biology, Seattle), transformed with a pRP4 plasmid expressing wild-type or mutant flagellin. The bacterial cells were transformed using electroporation at 2.5 kV, 200 Ω, and 25 μF in 10% glycerol. A single colony of transformed bacteria grown on LB agar plates containing 50 μg/ml ampicillin was selected and transferred to the motility test plates (LB media containing 0.3% agar, 1 mM IPTG, and 50 μg/ml ampicillin). The cultures were incubated overnight in an upright position at room temperature. Each plate was inoculated with a single colony of *S*. *typhimurium* expressing mutated flagellin and bacteria expressing wild-type SaTy as a control. The motility of the strains expressing the mutated flagellin was compared to the motility of the control strain transformed with wild-type SaTy.

### Activation of NAIP5/NLRC4 inflammasome

Immortalized wild type BMDMs from C57BL/6 mice and NLRP3-deficient mice (both gift of K. A. Fitzgerald; University of Massachusetts Medical school, Worcester, MA, USA) were cultured in DMEM supplemented with 10% FBS. NAIP5/NLRC4 activation assays were performed in serum-free DMEM. Cells were seeded at 1.5 x 10^5^ cells per well on 96 well plates and primed with ultra-pure LPS (100 ng/mL) for 6 hours for the stimulation of pro-IL-1β expression. Further, the growth medium was removed and wild type or mutant flagellin (3 μg/ml) mixed with DOTAP (1:5) in DMEM was added for 4 h. The concentration of secreted IL-1β was measured by ELISA (e-Bioscience) according to manufacturer’s instructions.

### Dynamic light scattering (DLS)

DLS data of the soluble flagellin species were acquired using a Zetasizer Nanoseries instrument (Malvern). The samples were centrifuged at 13000 rpm for 30 min to remove protein aggregates. Measurements were made at 20°C using automated settings, and 3 independent acquisitions of 10 measurements each were analyzed using the associated DTS nanoparticle-sizing software.

### Circular dichroism measurements (CD)

CD measurements were used to determine the secondary structure of soluble flagellins. The CD spectra were taken between 195 and 280 nm on a Chirascan CD spectrometer (Applied Photophysics) fused with nitrogen gas and equipped with a temperature controlled cuvette holder. A cell path length of 1 mm was used with concentrations of flagellins in the range of 0.1–0.5 mg/ml. All samples were dissolved in demi-water, and the results are the average of 3 spectra measured at 20°C.

### Flagellin sequence alignment

Flagellin amino acid sequences of *S*. *typhimurium* (SaTy, UniProt id. P06179), *S*. *marcescens* (SeMa, UniProt id. P13713), *S*. *dublin* (SaDu, UniProt id. Q06971), *H*. *pylori* (HePy, UniProt id. P0A0S1), and *C*. *jejuni* (CaJe, UniProt id. P22252) were aligned using ClustalW (http://embnet.vital-it.ch/software/ClustalW.html).

### Software and statistics

UCSF Chimera 1.6.2 software was used to generate the structural figures and to determine the distances among atoms (http://www.cgl.ucsf.edu/chimera/) [30]. Graphs were prepared with Origin 8.1 software (http://www.originlab.com/), and GraphPad Prism 5 (http://www.graphpad.com/) was used for statistics. A students’ unpaired two-tailed t-test was used for statistical comparison of the data.

## Supporting information

S1 Fig Fusion constructs between flagellin and TLR5 were expressed in comparable amounts in cells. (**A**) The expression of N-terminally FLAG-tagged chimeric constructs was analyzed by immunostaining. (**B**) Decreased activation of TLR5 in Fig 1D is not due to a decrease of TLR5 expression level. Cells were transfected with 100 ng of a plasmid expressing N-terminally AU1-tagged TLR5 (AU1-TLR5; lane 1) or 100 ng of the plasmid expressing AU1-TLR5 in combination with increasing amounts of plasmid expressing C-terminally AU1-tagged SFΔD0-TLR5 or a control plasmid pFLAG (900 ng).
(TIF)

S2 Fig Amino acid residues at the C-terminus of flagellin are crucial for TLR5 activation and NAIP5/NLRC4 imflammasome activation. (A) SDS-PAGE of purified recombinant proteins used for stimulation of TLR5. (B-E) Activation potential of selected alanine mutants of flagellin. The human lung epithelial A549 cells were stimulated with SaTy flagellin or mutants, and NF-κB-dependent SEAP activities were measured. (*Data are representative of two independent experiments*. *Bars represent the means of five biological replicates ±s*.*d*.; **p<0.*005*, ^*ns*^*p>0*.*05*). (F) HEK293 cells were transfected with a plasmid encoding hTLR5 (1 ng) or empty vector (as negative control) and stimulated with SaTy flagellin or mutants (0.8 or 1.6 ng/ml). After 18 h NF-κB-dependent firefly and Renilla luciferase activities were measured (Data are representative of three independent experiments. Bars represent the means of four biological replicates ±s.d.;**p<0.005). (G) Amino acid alignment of C-terminal sequences of *S*. *typhimurium* flagellin (SaTy) and *H*. *pylori* flagellin (HePy). Amino acid residues selected for substitution from SaTy to HePy counterparts are indicated with a line (TEVS to EESA and QQA to VQS). (**H,I**) Activation of NAIP5/NLRC4 inflammasome wild type (Mϕwt) (H) and NLRP3 knock out (MϕNLRP3^-^) (I) macrophages by wild type flagellin and the VLSLL_A mutant. Macrophages were primed with LPS for 6 h and stimulated with SaTy or VLSLL_A mutant (3 μg/ml) for 4 h. Activation of NAIP5/NLRC4 inflammasome was determined by measuring IL-1β expression in media using ELISA.
(TIF)

S3 Fig Molecular model of the hTLR5 ectodomain (aa 21–639) and the full-length *S*. *typhimurium* flagellin superimposed on drTLR5-N14_VLR_/sdFliC-ΔD0 from the crystal structure. (A) hTLR5 ectodomain (magenta and cyan), *S*. *typhimurium* flagellin (grey), drTLR5-N14_VLR_ (yellow) from the crystal structure drTLR5-N14_VLR_/sdFliC-ΔD0. C-termini of TLR5^ECD^ are 55 Å apart. The membrane into which TLR5 is inserted prevents a linear extension of the D0 relative to D1. For docking, we used the model of hTLR5 built with I-TASSER, the crystal structure of TLR5-N14 with a D0 deletion variant of sdFliC [14], and a 3D structure of *S*. *typhimurium* flagellin (PDB code 1UCU) [6]. The hTLR5 ectodomain structural model was generated using the I-TASSER server (http://zhanglab.ccmb.med.umich.edu/I-TASSER/) [31,32]. (B) Molecular model of the heterodimer of the hTLR5 ectodomain (aa 21–639) and the full-length *S*. *typhimurium* flagellin. Amino acid residues with an effect on TLR5 activation are highlighted.
(TIF)

S4 Fig Tethered inactive flagellin substantially restores activation of TLR5. HEK293 cells transfected with a plasmid encoding hTLR5 (5 ng) were stimulated with increasing concentrations (0–50 ng/ml) of recombinant proteins (SaTy flagellin, SFΔD0, or dimSFΔD0) and NF-κB-dependent firefly and *Renilla* luciferase activities were measured. (*Data are representative of three independent experiments*. *Points and bars represent the means of four biological replicates ±s*.*d*.; **p<0.*005*, ^*ns*^*p>0*.*05*).
(TIF)

S5 Fig The D0 domain of *H*. *pylori* flagellin contributes to the evasion of TLR5 recognition. (**A**) Isolated proteins were assessed for purity by SDS-PAGE. (**B**) A chimeric flagellin SaTy-CD0(HePy) is inefficient in promoting signaling. (**C**) A chimeric flagellin of *H*. *pylori* with exchanged D0 domains of *S*. *typhimurium* (HePy-CD0(SaTy)) fails to activate TLR5. (**D**) Selected counterpart mutations of chimeric protein SaTy-CD0(HePy)^mut^ partially restored the activation efficiency of flagellin to TLR5. (**B-D**) HEK293 cells transfected with a plasmid encoding hTLR5 (5 ng) or a vector (5 ng, as a negative control) were stimulated with SaTy flagellin or chimeric proteins. NF-κB-dependent firefly and *Renilla* luciferase activities were measured, and normalized luciferase activity is shown. (*Data are representative of three independent experiments*. *Bars represent the means of 4 biological replicates ±s*.*d*.; **p<0.*005*, ^*ns*^*p>0*.*05*).
(TIF)

S1 Table Related to experimental procedure. Sequences of chimeric proteins used in the present study.
(DOCX)
