# Supplementary figures and images for: The role of the C-terminal D0 domain of flagellin in activation of Toll like receptor 5
Source: PLoS Pathog. 2017 Aug 21;13(8):e1006574. doi: 10.1371/journal.ppat.1006574 (PMC5578693; doi:10.1371/journal.ppat.1006574)

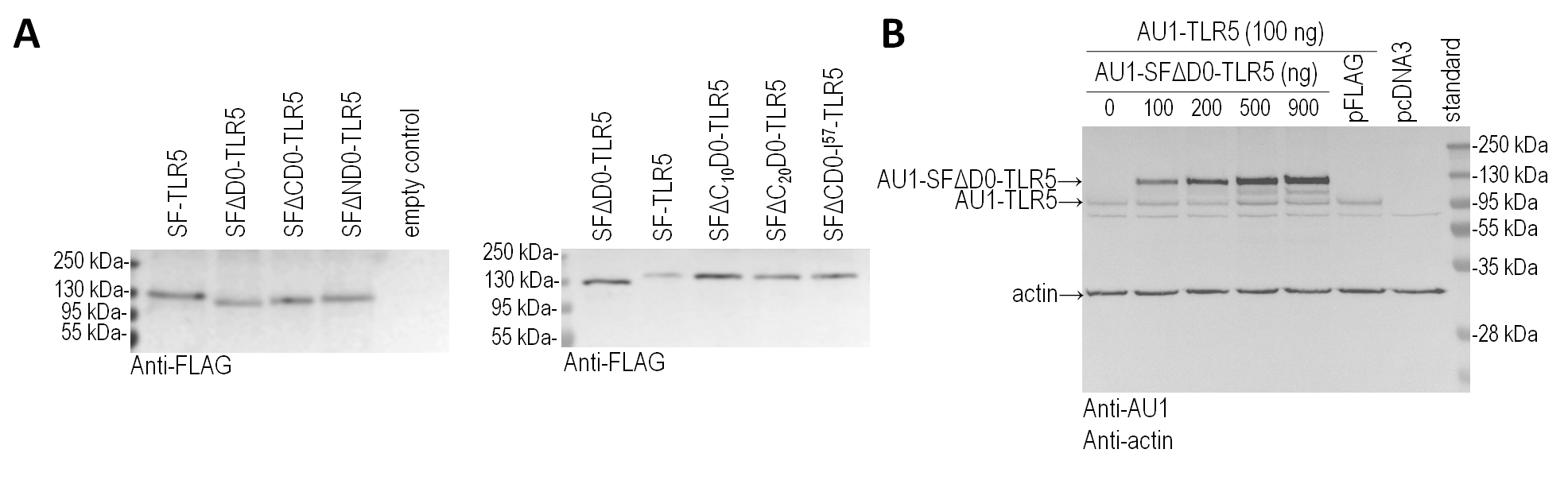

Supplement: S1 Fig — (A) The expression of N-terminally FLAG-tagged chimeric constructs was analyzed by immunostaining. (B) Decreased activation of TLR5 in Fig 1D is not due to a decrease of TLR5 expression level. Cells were transfected with 100 ng of a plasmid expressing N-terminally AU1-tagged TLR5 (AU1-TLR5; lane 1) or 100 ng of the plasmid expressing AU1-TLR5 in combination with increasing amounts of plasmid expressing C-terminally AU1-tagged SFΔD0-TLR5 or a control plasmid pFLAG (900 ng). (TIF) [file ppat.1006574.s001.tif]

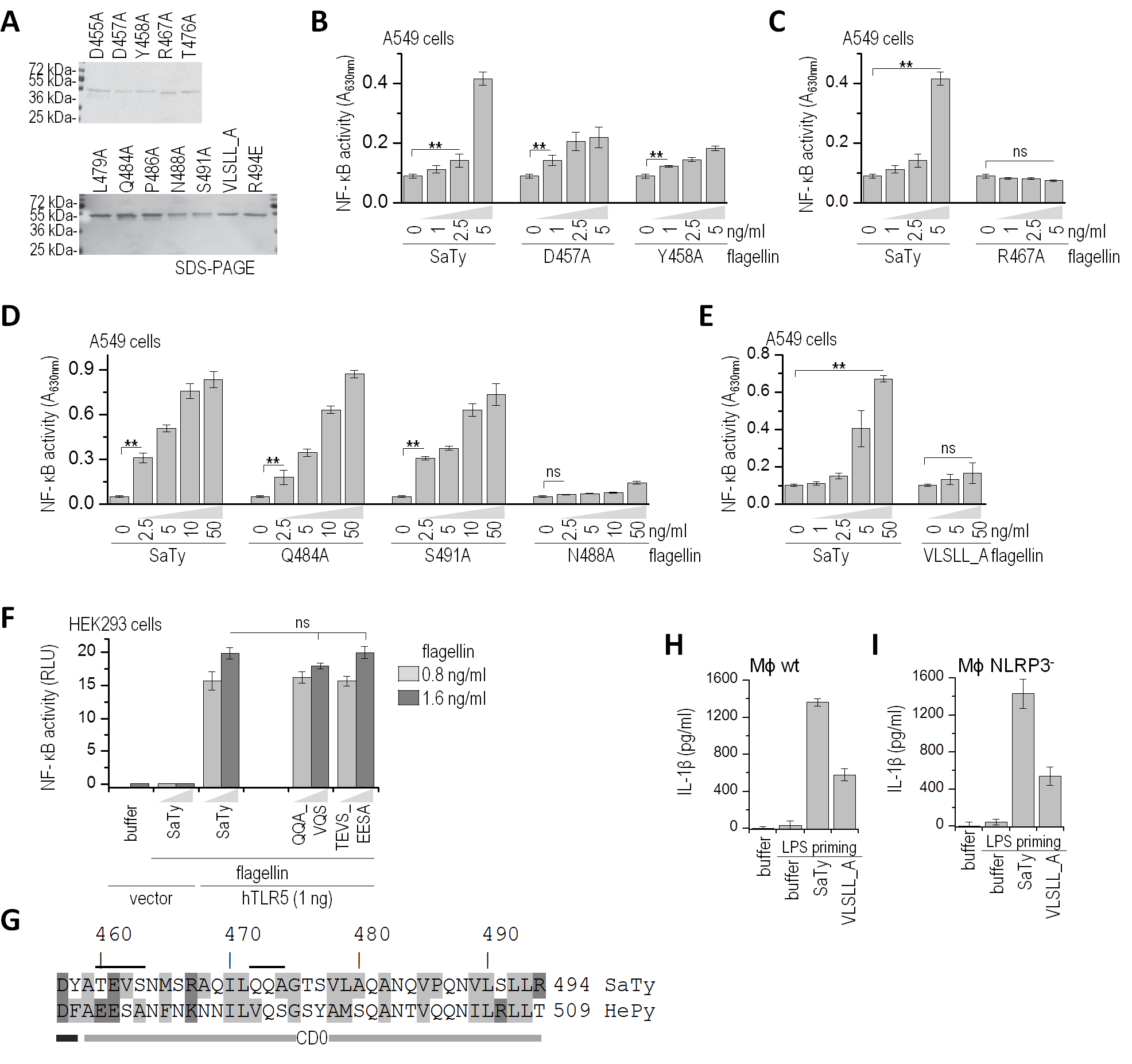

Supplement: S2 Fig — (A) SDS-PAGE of purified recombinant proteins used for stimulation of TLR5. (B-E) Activation potential of selected alanine mutants of flagellin. The human lung epithelial A549 cells were stimulated with SaTy flagellin or mutants, and NF-κB-dependent SEAP activities were measured. (Data are representative of two independent experiments. Bars represent the means of five biological replicates ±s.d.; **p<0.005, nsp>0.05). (F) HEK293 cells were transfected with a plasmid encoding hTLR5 (1 ng) or empty vector (as negative control) and stimulated with SaTy flagellin or mutants (0.8 or 1.6 ng/ml). After 18 h NF-κB-dependent firefly and Renilla luciferase activities were measured (Data are representative of three independent experiments. Bars represent the means of four biological replicates ±s.d.;**p<0.005). (G) Amino acid alignment of C-terminal sequences of S. typhimurium flagellin (SaTy) and H. pylori flagellin (HePy). Amino acid residues selected for substitution from SaTy to HePy counterparts are indicated with a line (TEVS to EESA and QQA to VQS). (H,I) Activation of NAIP5/NLRC4 inflammasome wild type (Mϕwt) (H) and NLRP3 knock out (MϕNLRP3-) (I) macrophages by wild type flagellin and the VLSLL_A mutant. Macrophages were primed with LPS for 6 h and stimulated with SaTy or VLSLL_A mutant (3 μg/ml) for 4 h. Activation of NAIP5/NLRC4 inflammasome was determined by measuring IL-1β expression in media using ELISA. (TIF) [file ppat.1006574.s002.tif]

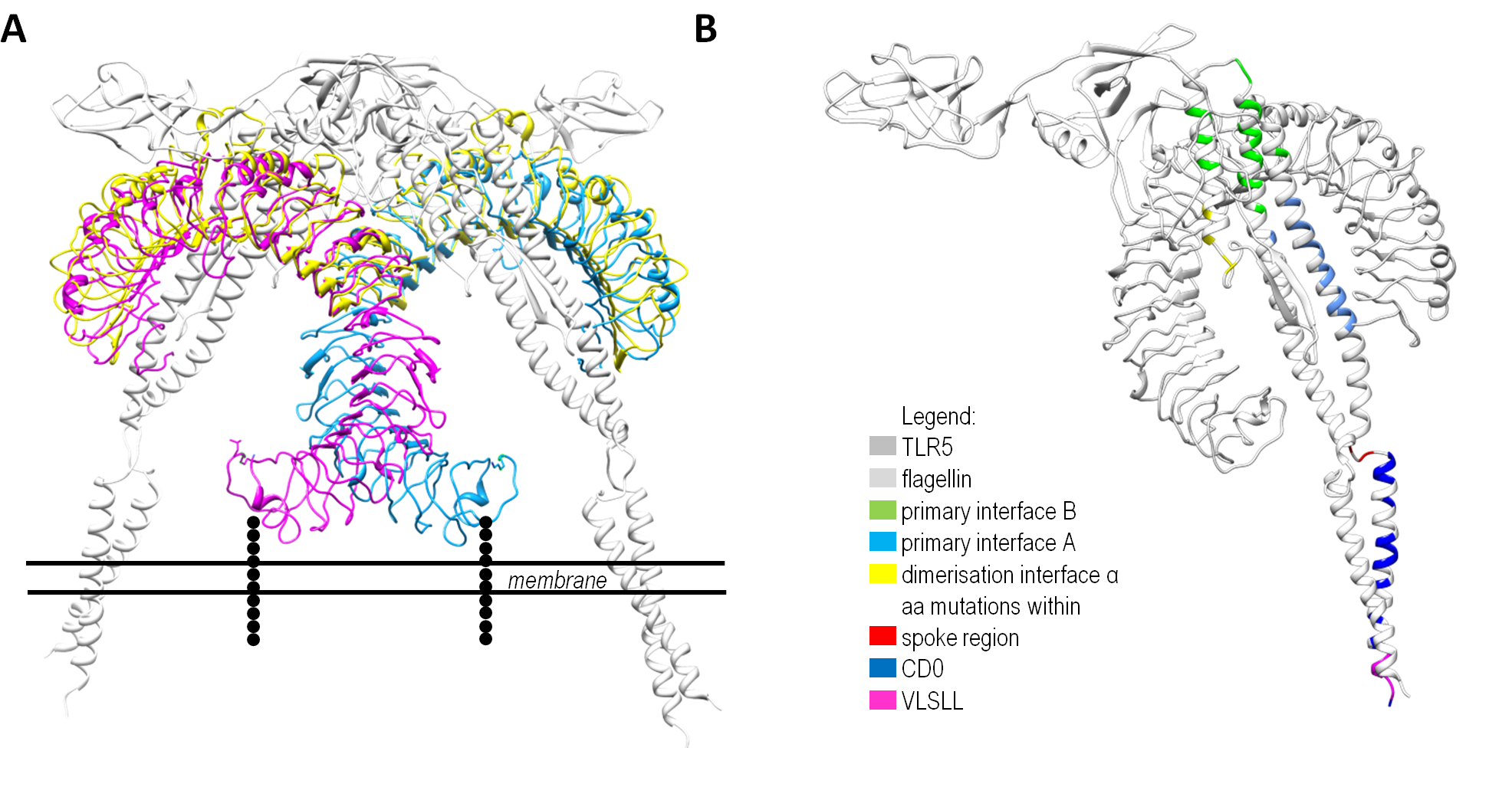

Supplement: S3 Fig — (A) hTLR5 ectodomain (magenta and cyan), S. typhimurium flagellin (grey), drTLR5-N14VLR (yellow) from the crystal structure drTLR5-N14VLR/sdFliC-ΔD0. C-termini of TLR5ECD are 55 Å apart. The membrane into which TLR5 is inserted prevents a linear extension of the D0 relative to D1. For docking, we used the model of hTLR5 built with I-TASSER, the crystal structure of TLR5-N14 with a D0 deletion variant of sdFliC [14], and a 3D structure of S. typhimurium flagellin (PDB code 1UCU) [6]. The hTLR5 ectodomain structural model was generated using the I-TASSER server (http://zhanglab.ccmb.med.umich.edu/I-TASSER/) [31,32]. (B) Molecular model of the heterodimer of the hTLR5 ectodomain (aa 21–639) and the full-length S. typhimurium flagellin. Amino acid residues with an effect on TLR5 activation are highlighted. (TIF) [file ppat.1006574.s003.tif]

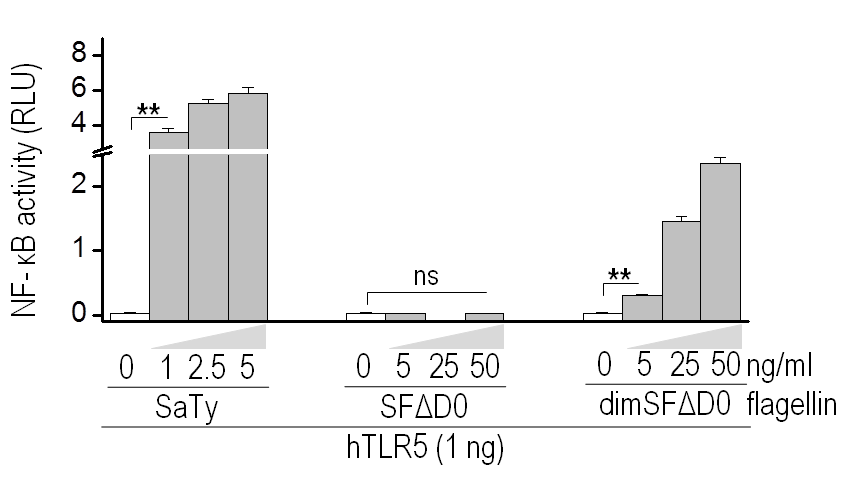

Supplement: S4 Fig — HEK293 cells transfected with a plasmid encoding hTLR5 (5 ng) were stimulated with increasing concentrations (0–50 ng/ml) of recombinant proteins (SaTy flagellin, SFΔD0, or dimSFΔD0) and NF-κB-dependent firefly and Renilla luciferase activities were measured. (Data are representative of three independent experiments. Points and bars represent the means of four biological replicates ±s.d.; **p<0.005, nsp>0.05). (TIF) [file ppat.1006574.s004.tif]

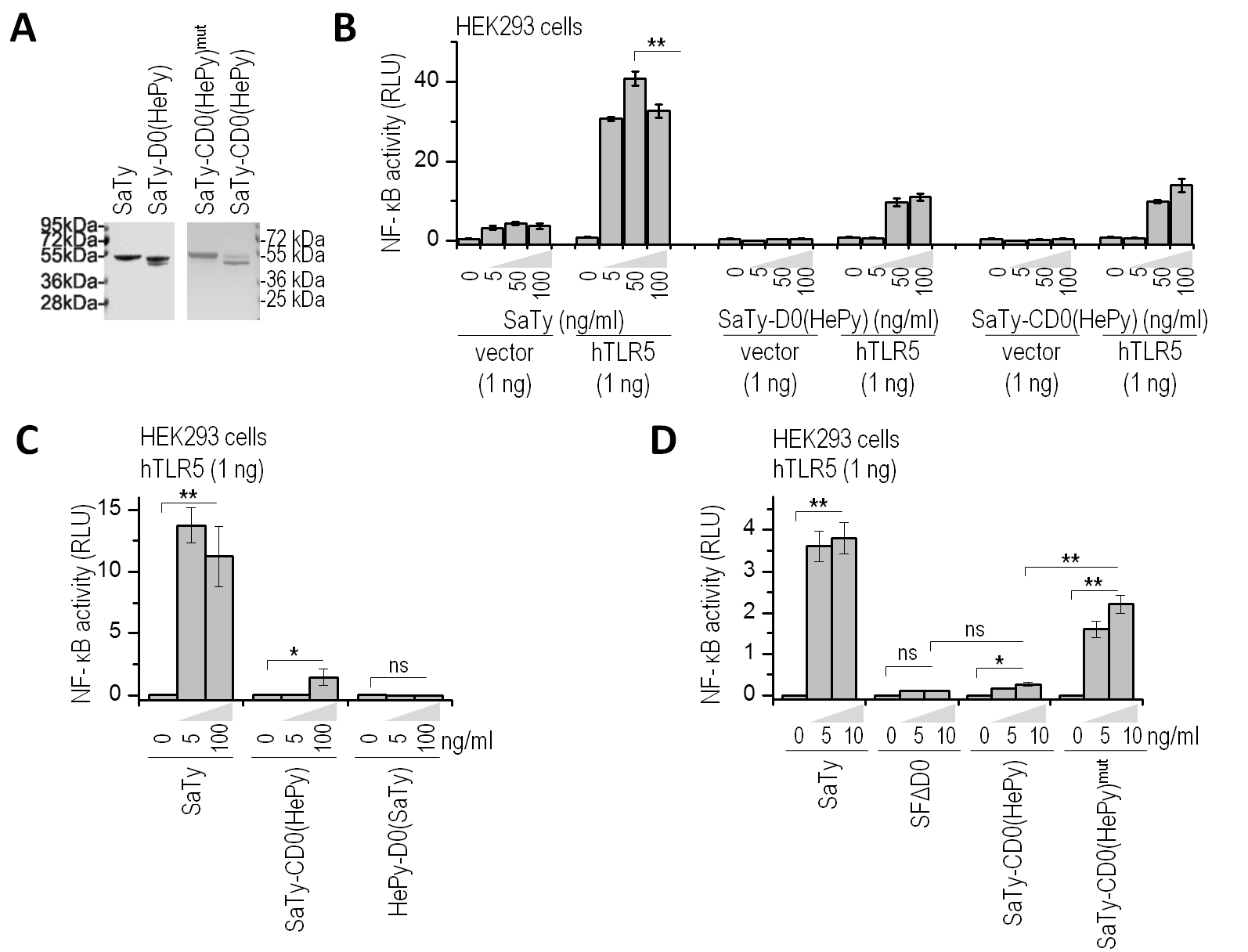

Supplement: S5 Fig — (A) Isolated proteins were assessed for purity by SDS-PAGE. (B) A chimeric flagellin SaTy-CD0(HePy) is inefficient in promoting signaling. (C) A chimeric flagellin of H. pylori with exchanged D0 domains of S. typhimurium (HePy-CD0(SaTy)) fails to activate TLR5. (D) Selected counterpart mutations of chimeric protein SaTy-CD0(HePy)mut partially restored the activation efficiency of flagellin to TLR5. (B-D) HEK293 cells transfected with a plasmid encoding hTLR5 (5 ng) or a vector (5 ng, as a negative control) were stimulated with SaTy flagellin or chimeric proteins. NF-κB-dependent firefly and Renilla luciferase activities were measured, and normalized luciferase activity is shown. (Data are representative of three independent experiments. Bars represent the means of 4 biological replicates ±s.d.; **p<0.005, nsp>0.05). (TIF) [file ppat.1006574.s005.tif]
