# Supplementary material for: The role of the C-terminal D0 domain of flagellin in activation of Toll like receptor 5
Source: PLoS Pathog. 2017 Aug 21;13(8):e1006574. doi: 10.1371/journal.ppat.1006574 (PMC5578693; doi:10.1371/journal.ppat.1006574)
Supplement: S1 Table — Sequences of chimeric proteins used in the present study. (DOCX) [file ppat.1006574.s006.docx]

**S1 Table.** *Related to Experimental Procedure.* Sequences of chimeric proteins used in the present study.

| SFΔD0 | Short flagellin lacking the D0 domain (SFΔD0) contains the N-terminal D1 (43-176) and C-terminal D1 (398-456) domains of SaTy linked via a 13 aa linker. The D1 domains of SaTy were PCR amplified and linked via a PCR overlap extension method. SFΔD0 was cloned into the pET19b vector using the Gibson assembly method. |
| --- | --- |
| TMGHHHHHHHHHHSSGHIDDDDKHMYDDAAGQAIANRFTANIKGLTQASRNANDGISIAQTTEGALNEINNNLQRVRELAVQSANSTNSQSDLDSIQAEITQRLNEIDRVSGQTQFNGVKVLAQDNTLTIQVGANDGETIDIDLKQINSQTLGLDTLNVQISGGGGGILDSMGAEAAATTTENPLQKIDAALAQVDTLRSDLGAVQNRFNSAITNLGNTVNNLTSARSRIED#GS | |
| dimSFΔD0 | Two SFΔD0 were PCR amplified, linked together via a 27 aa linker and cloned into the pET19b vector containing an N-terminal His-tag using the Gibson assembly method. |
| TMGHHHHHHHHHHSSGHIDDDDKHMMDDAAGQAIANRFTANIKGLTQASRNANDGISIAQTTEGALNEINNNLQRVRELAVQSANSTNSQSDLDSIQAEITQRLNEIDRVSGQTQFNGVKVLAQDNTLTIQVGANDGETIDIDLKQINSQTLGLDTLNVQISGGGGGILDSMGAEAAATTTENPLQKIDAALAQVDTLRSDLGAVQNRFNSAITNLGNTVNNLTSARSRIEDGSEGKSSGSGSESKVTDSGSETGSSDDAAGQAIANRFTANIKGLTQASRNANDGISIAQTTEGALNEINNNLQRVRELAVQSANSTNSQSDLDSIQAEITQRLNEIDRVSGQTQFNGVKVLAQDNTLTIQVGANDGETIDIDLKQINSQTLGLDTLNVQISGGGGGILDSMGAEAAATTTENPLQKIDAALAQVDTLRSDLGAVQNRFNSAITNLGNTVNNLTSARSRIED#GS | |
| SaTy-D0(HePy) | SaTy-D0(HePy) contains the D0 domain from HePy (1-42; 477-511) and D1, D2 and D3 domains from SaTy (43-461). The construct was prepared with Gibson assembly and cloned into the pET19b vector containing an N-terminal His-tag. |
| MGHHHHHHHHHHSSGHIDDDDKHMLEMAFQVNTNINAMNAHVQSALTQNALKTSLERLSSGLRINKAADDAAGQAIANRFTANIKGLTQASRNANDGISIAQTTEGALNEINNNLQRVRELAVQSANSTNSQSDLDSIQAEITQRLNEIDRVSGQTQFNGVKVLAQDNTLTIQVGANDGETIDIDLKQINSQTLGLDTLNVQQKYKVSDTAATVTGYADTTIALDNSTFKASATGLGGTDQKIDGDLKFDDTTGKYYAKVTVTGGTGKDGYYEVSVDKTNGEVTLAGGATSPLTGGLPATATEDVKNVQVANADLTEAKAALTAAGVTGTASVVKMSYTDNNGKTIDGGLAVKVGDDYYSATQNKDGSISINTTKYTADDGTSKTALNKLGGADGKTEVVSIGGKTYAASKAEGHNFKAQPDLAEAAATTTENPLQKIDAALAQVDTLRSDLGAVQNRFNSAITNLGNTVNNLTSARSRIEDSDYATESANFNKNNILVQSGSYAMSQANTVQQNILRLLT#GS | |
| SaTy-CD0(HePy) | SaTy-CD0(HePy) contains SaTy flagellin (1-454) with the C-terminal spoke region and D0 domain of HePy (471-511). The construct was prepared with Gibson assembly and cloned into the pET19b vector containing an N-terminal His-tag. |
| MGHHHHHHHHHHSSGHIDDDDKHMLEMAQVINTNSLSLLTQNNLNKSQSALGTAIERLSSGLRINSAKDDAAGQAIANRFTANIKGLTQASRNANDGISIAQTTEGALNEINNNLQRVRELAVQSANSTNSQSDLDSIQAEITQRLNEIDRVSGQTQFNGVKVLAQDNTLTIQVGANDGETIDIDLKQINSQTLGLDTLNVQQKYKVSDTAATVTGYADTTIALDNSTFKASATGLGGTDQKIDGDLKFDDTTGKYYAKVTVTGGTGKDGYYEVSVDKTNGEVTLAGGATSPLTGGLPATATEDVKNVQVANADLTEAKAALTAAGVTGTASVVKMSYTDNNGKTIDGGLAVKVGDDYYSATQNKDGSISINTTKYTADDGTSKTALNKLGGADGKTEVVSIGGKTYAASKAEGHNFKAQPDLAEAAATTTENPLQKIDAALAQVDTLRSDLGAVQNRFNSAITNLGNTVNNLTSARSRIRDVDFAEESANFNKNNILVQSGSYAMSQANTVQQNILRLLT#GS | |
| SaTy-CD0(HePy)^mut^ | SaTy-CD0(HePy)^mut^ contains SaTy flagellin (1-454) with the C-terminal spoke region and D0 domain of HePy (471-511) containing point mutations reversing amino acid residues back to corresponding SaTy counterparts (highlighted in red), thus restoring the protein’s activation potential. The construct was prepared with Gibson assembly and cloned into the pET19b vector containing an N-terminal His-tag. |
| MGHHHHHHHHHHSSGHIDDDDKHMLEMAQVINTNSLSLLTQNNLNKSQSALGTAIERLSSGLRINSAKDDAAGQAIANRFTANIKGLTQASRNANDGISIAQTTEGALNEINNNLQRVRELAVQSANSTNSQSDLDSIQAEITQRLNEIDRVSGQTQFNGVKVLAQDNTLTIQVGANDGETIDIDLKQINSQTLGLDTLNVQQKYKVSDTAATVTGYADTTIALDNSTFKASATGLGGTDQKIDGDLKFDDTTGKYYAKVTVTGGTGKDGYYEVSVDKTNGEVTLAGGATSPLTGGLPATATEDVKNVQVANADLTEAKAALTAAGVTGTASVVKMSYTDNNGKTIDGGLAVKVGDDYYSATQNKDGSISINTTKYTADDGTSKTALNKLGGADGKTEVVSIGGKTYAASKAEGHNFKAQPDLAEAAATTTENPLQKIDAALAQVDTLRSDLGAVQNRFNSAITNLGNTVNNLTSARSRIEDVDFATESANFNKNNILVQSGSSAMSQANTVQQNILSLLR#GS | |
| SF-TLR5 | SF-TLR5 contains short flagellin composed of the D0 and D1 domains (aa 1 - aa 176, a 13 aa linker and aa 398 – aa 494), linked through a 27 amino acid linker to hTLR5 (21-858) with an AU1-tag at the C-terminal end. The construct was inserted into the pFLAG-CMV3 vector downstream of an N-terminal preprotrypsin leader sequence followed by an N-terminal FLAG-tag. |
| DYKDDDDKAQVINTNSLSLLTQNNLNKSQSALGTAIERLSSGLRINSAKDDAAGQAIANRFTANIKGLTQASRNANDGISIAQTTEGALNEINNNLQRVRELAVQSANSTNSQSDLDSIQAEITQRLNEIDRVSGQTQFNGVKVLAQDNTLTIQVGANDGETIDIDLKQINSQTLGLDTLNVQISGGGGGILDSMGAEAAATTTENPLQKIDAALAQVDTLRSDLGAVQNRFNSAITNLGNTVNNLTSARSRIEDSDYATEVSNMSRAQILQQAGTSVLAQANQVPQNVLSLLRGSEGKSSGSGSESKVTDSGSETGSSGSIPSCSFDGRIAFYRFCNLTQVPQVLNTTERLLLSFNYIRTVTASSFPFLEQLQLLELGSQYTPLTIDKEAFRNLPNLRILDLGSSKIYFLHPDAFQGLFHLFELRLYFCGLSDAVLKDGYFRNLKALTRLDLSKNQIRSLYLHPSFGKLNSLKSIDFSSNQIFLVCEHELEPLQGKTLSFFSLAANSLYSRVSVDWGKCMNPFRNMVLEILDVSGNGWTVDITGNFSNAISKSQAFSLILAHHIMGAGFGFHNIKDPDQNTFAGLARSSVRHLDLSHGFVFSLNSRVFETLKDLKVLNLAYNKINKIADEAFYGLDNLQVLNLSYNLLGELYSSNFYGLPKVAYIDLQKNHIAIIQDQTFKFLEKLQTLDLRDNALTTIHFIPSIPDIFLSGNKLVTLPKINLTANLIHLSENRLENLDILYFLLRVPHLQILILNQNRFSSCSGDQTPSENPSLEQLFLGENMLQLAWETELCWDVFEGLSHLQVLYLNHNYLNSLPPGVFSHLTALRGLSLNSNRLTVLSHNDLPANLEILDISRNQLLAPNPDVFVSLSVLDITHNKFICECELSTFINWLNHTNVTIAGPPADIYCVYPDSLSGVSLFSLSTEGCDEEEVLKSLKFSLFIVCTVTLTLFLMTILTVTKFRGFCFICYKTAQRLVFKDHPQGTEPDMYKYDAYLCFSSKDFTWVQNALLKHLDTQYSDQNRFNLCFEERDFVPGENRIANIQDAIWNSRKIVCLVSRHFLRDGWCLEAFSYAQGRCLSDLNSALIMVVVGSLSQYQLMKHQSIRGFVQKQQYLRWPEDLQDVGWFLHKLSQQILKKEKEKKKDNNIPLQTVATISDTYRYI#RS | |
| SFΔND0-TLR5 | SFΔND0-TLR5 contains short flagellin lacking the N-terminal D0 domain (aa 43 - aa 176, a 13 aa linker and aa 398 – aa 494), linked through a 27 amino acid linker to hTLR5 (21-858) with an AU1-tag at the C-terminal end. The construct was inserted into the pFLAG-CMV3 vector downstream of an N-terminal preprotrypsin leader sequence followed by an N-terminal FLAG-tag. |
| DYKDDDDK________________________________SSGLRINSAKDDAAGQAIANRFTANIKGLTQASRNANDGISIAQTTEGALNEINNNLQRVRELAVQSANSTNSQSDLDSIQAEITQRLNEIDRVSGQTQFNGVKVLAQDNTLTIQVGANDGETIDIDLKQINSQTLGLDTLNVQISGGGGGILDSMGAEAAATTTENPLQKIDAALAQVDTLRSDLGAVQNRFNSAITNLGNTVNNLTSARSRIEDSDYATEVSNMSRAQILQQAGTSVLAQANQVPQNVLSLLRGSEGKSSGSGSESKVTDSGSETGSSGSIPSCSFDGRIAFYRFCNLTQVPQVLNTTERLLLSFNYIRTVTASSFPFLEQLQLLELGSQYTPLTIDKEAFRNLPNLRILDLGSSKIYFLHPDAFQGLFHLFELRLYFCGLSDAVLKDGYFRNLKALTRLDLSKNQIRSLYLHPSFGKLNSLKSIDFSSNQIFLVCEHELEPLQGKTLSFFSLAANSLYSRVSVDWGKCMNPFRNMVLEILDVSGNGWTVDITGNFSNAISKSQAFSLILAHHIMGAGFGFHNIKDPDQNTFAGLARSSVRHLDLSHGFVFSLNSRVFETLKDLKVLNLAYNKINKIADEAFYGLDNLQVLNLSYNLLGELYSSNFYGLPKVAYIDLQKNHIAIIQDQTFKFLEKLQTLDLRDNALTTIHFIPSIPDIFLSGNKLVTLPKINLTANLIHLSENRLENLDILYFLLRVPHLQILILNQNRFSSCSGDQTPSENPSLEQLFLGENMLQLAWETELCWDVFEGLSHLQVLYLNHNYLNSLPPGVFSHLTALRGLSLNSNRLTVLSHNDLPANLEILDISRNQLLAPNPDVFVSLSVLDITHNKFICECELSTFINWLNHTNVTIAGPPADIYCVYPDSLSGVSLFSLSTEGCDEEEVLKSLKFSLFIVCTVTLTLFLMTILTVTKFRGFCFICYKTAQRLVFKDHPQGTEPDMYKYDAYLCFSSKDFTWVQNALLKHLDTQYSDQNRFNLCFEERDFVPGENRIANIQDAIWNSRKIVCLVSRHFLRDGWCLEAFSYAQGRCLSDLNSALIMVVVGSLSQYQLMKHQSIRGFVQKQQYLRWPEDLQDVGWFLHKLSQQILKKEKEKKKDNNIPLQTVATISDTYRYI#RS | |
| SFΔCD0-TLR5 | SFΔCD0-TLR5 contains short flagellin lacking the C-terminal D0 domain (aa 1 - aa 176, a 13 aa linker and aa 398 – aa 458), linked through a 27 amino acid linker to hTLR5 (21-858) with an AU1-tag at the C-terminal end. The construct was inserted into the pFLAG-CMV3 vector downstream of an N-terminal preprotrypsin leader sequence followed by an N-terminal FLAG-tag. |
| DYKDDDDKAQVINTNSLSLLTQNNLNKSQSALGTAIERLSSGLRINSAKDDAAGQAIANRFTANIKGLTQASRNANDGISIAQTTEGALNEINNNLQRVRELAVQSANSTNSQSDLDSIQAEITQRLNEIDRVSGQTQFNGVKVLAQDNTLTIQVGANDGETIDIDLKQINSQTLGLDTLNVQISGGGGGILDSMGAEAAATTTENPLQKIDAALAQVDTLRSDLGAVQNRFNSAITNLGNTVNNLTSARSRIEDSDY_______________________________________GSEGKSSGSGSESKVTDSGSETGSSGSIPSCSFDGRIAFYRFCNLTQVPQVLNTTERLLLSFNYIRTVTASSFPFLEQLQLLELGSQYTPLTIDKEAFRNLPNLRILDLGSSKIYFLHPDAFQGLFHLFELRLYFCGLSDAVLKDGYFRNLKALTRLDLSKNQIRSLYLHPSFGKLNSLKSIDFSSNQIFLVCEHELEPLQGKTLSFFSLAANSLYSRVSVDWGKCMNPFRNMVLEILDVSGNGWTVDITGNFSNAISKSQAFSLILAHHIMGAGFGFHNIKDPDQNTFAGLARSSVRHLDLSHGFVFSLNSRVFETLKDLKVLNLAYNKINKIADEAFYGLDNLQVLNLSYNLLGELYSSNFYGLPKVAYIDLQKNHIAIIQDQTFKFLEKLQTLDLRDNALTTIHFIPSIPDIFLSGNKLVTLPKINLTANLIHLSENRLENLDILYFLLRVPHLQILILNQNRFSSCSGDQTPSENPSLEQLFLGENMLQLAWETELCWDVFEGLSHLQVLYLNHNYLNSLPPGVFSHLTALRGLSLNSNRLTVLSHNDLPANLEILDISRNQLLAPNPDVFVSLSVLDITHNKFICECELSTFINWLNHTNVTIAGPPADIYCVYPDSLSGVSLFSLSTEGCDEEEVLKSLKFSLFIVCTVTLTLFLMTILTVTKFRGFCFICYKTAQRLVFKDHPQGTEPDMYKYDAYLCFSSKDFTWVQNALLKHLDTQYSDQNRFNLCFEERDFVPGENRIANIQDAIWNSRKIVCLVSRHFLRDGWCLEAFSYAQGRCLSDLNSALIMVVVGSLSQYQLMKHQSIRGFVQKQQYLRWPEDLQDVGWFLHKLSQQILKKEKEKKKDNNIPLQTVATISDTYRYI#RS | |
| SFΔCD0-l^57^-TLR5 | SFΔCD0-TLR5 contains short flagellin lacking the C-terminal D0 domain (aa 1 - aa 176, a 13 aa linker and aa 398 – aa 458), linked through a 57 amino acid linker to hTLR5 (21-858) with an AU1-tag at the C-terminal end. The construct was inserted into the pFLAG-CMV3 vector downstream of an N-terminal preprotrypsin leader sequence followed by an N-terminal FLAG-tag. |
| DYKDDDDKAQVINTNSLSLLTQNNLNKSQSALGTAIERLSSGLRINSAKDDAAGQAIANRFTANIKGLTQASRNANDGISIAQTTEGALNEINNNLQRVRELAVQSANSTNSQSDLDSIQAEITQRLNEIDRVSGQTQFNGVKVLAQDNTLTIQVGANDGETIDIDLKQINSQTLGLDTLNVQISGGGGGILDSMGAEAAATTTENPLQKIDAALAQVDTLRSDLGAVQNRFNSAITNLGNTVNNLTSARSRIEDSDY_______________________________________GSEGKSSGSGSESKVTDSGSETGSSGSEKGSGSEKVDSTGSGSSEKTDVSESGSGSGIPSCSFDGRIAFYRFCNLTQVPQVLNTTERLLLSFNYIRTVTASSFPFLEQLQLLELGSQYTPLTIDKEAFRNLPNLRILDLGSSKIYFLHPDAFQGLFHLFELRLYFCGLSDAVLKDGYFRNLKALTRLDLSKNQIRSLYLHPSFGKLNSLKSIDFSSNQIFLVCEHELEPLQGKTLSFFSLAANSLYSRVSVDWGKCMNPFRNMVLEILDVSGNGWTVDITGNFSNAISKSQAFSLILAHHIMGAGFGFHNIKDPDQNTFAGLARSSVRHLDLSHGFVFSLNSRVFETLKDLKVLNLAYNKINKIADEAFYGLDNLQVLNLSYNLLGELYSSNFYGLPKVAYIDLQKNHIAIIQDQTFKFLEKLQTLDLRDNALTTIHFIPSIPDIFLSGNKLVTLPKINLTANLIHLSENRLENLDILYFLLRVPHLQILILNQNRFSSCSGDQTPSENPSLEQLFLGENMLQLAWETELCWDVFEGLSHLQVLYLNHNYLNSLPPGVFSHLTALRGLSLNSNRLTVLSHNDLPANLEILDISRNQLLAPNPDVFVSLSVLDITHNKFICECELSTFINWLNHTNVTIAGPPADIYCVYPDSLSGVSLFSLSTEGCDEEEVLKSLKFSLFIVCTVTLTLFLMTILTVTKFRGFCFICYKTAQRLVFKDHPQGTEPDMYKYDAYLCFSSKDFTWVQNALLKHLDTQYSDQNRFNLCFEERDFVPGENRIANIQDAIWNSRKIVCLVSRHFLRDGWCLEAFSYAQGRCLSDLNSALIMVVVGSLSQYQLMKHQSIRGFVQKQQYLRWPEDLQDVGWFLHKLSQQILKKEKEKKKDNNIPLQTVATISDTYRYI#RS | |
| SFΔC_20_D0-TLR5 | SFΔCD0-TLR5 contains short flagellin lacking the C-terminal D0 domain (aa 1 - aa 176, a 13 aa linker and aa 398 – aa 458), linked through a 27 amino acid linker to hTLR5 (21-858) with an AU1-tag at the C-terminal end. The construct was inserted into the pFLAG-CMV3 vector downstream of an N-terminal preprotrypsin leader sequence followed by an N-terminal FLAG-tag. |
| DYKDDDDKAQVINTNSLSLLTQNNLNKSQSALGTAIERLSSGLRINSAKDDAAGQAIANRFTANIKGLTQASRNANDGISIAQTTEGALNEINNNLQRVRELAVQSANSTNSQSDLDSIQAEITQRLNEIDRVSGQTQFNGVKVLAQDNTLTIQVGANDGETIDIDLKQINSQTLGLDTLNVQISGGGGGILDSMGAEAAATTTENPLQKIDAALAQVDTLRSDLGAVQNRFNSAITNLGNTVNNLTSARSRIEDSDYATEVSNMSRAQILQQA____________________GSEGKSSGSGSESKVTDSGSETGSSGSIPSCSFDGRIAFYRFCNLTQVPQVLNTTERLLLSFNYIRTVTASSFPFLEQLQLLELGSQYTPLTIDKEAFRNLPNLRILDLGSSKIYFLHPDAFQGLFHLFELRLYFCGLSDAVLKDGYFRNLKALTRLDLSKNQIRSLYLHPSFGKLNSLKSIDFSSNQIFLVCEHELEPLQGKTLSFFSLAANSLYSRVSVDWGKCMNPFRNMVLEILDVSGNGWTVDITGNFSNAISKSQAFSLILAHHIMGAGFGFHNIKDPDQNTFAGLARSSVRHLDLSHGFVFSLNSRVFETLKDLKVLNLAYNKINKIADEAFYGLDNLQVLNLSYNLLGELYSSNFYGLPKVAYIDLQKNHIAIIQDQTFKFLEKLQTLDLRDNALTTIHFIPSIPDIFLSGNKLVTLPKINLTANLIHLSENRLENLDILYFLLRVPHLQILILNQNRFSSCSGDQTPSENPSLEQLFLGENMLQLAWETELCWDVFEGLSHLQVLYLNHNYLNSLPPGVFSHLTALRGLSLNSNRLTVLSHNDLPANLEILDISRNQLLAPNPDVFVSLSVLDITHNKFICECELSTFINWLNHTNVTIAGPPADIYCVYPDSLSGVSLFSLSTEGCDEEEVLKSLKFSLFIVCTVTLTLFLMTILTVTKFRGFCFICYKTAQRLVFKDHPQGTEPDMYKYDAYLCFSSKDFTWVQNALLKHLDTQYSDQNRFNLCFEERDFVPGENRIANIQDAIWNSRKIVCLVSRHFLRDGWCLEAFSYAQGRCLSDLNSALIMVVVGSLSQYQLMKHQSIRGFVQKQQYLRWPEDLQDVGWFLHKLSQQILKKEKEKKKDNNIPLQTVATISDTYRYI#RS | |
| SFΔC_10_D0-TLR5 | SFΔCD0-TLR5 contains short flagellin lacking the C-terminal D0 domain (aa 1 - aa 176, a 13 aa linker and aa 398 – aa 468), linked through a 27 amino acid linker to hTLR5 (21-858) with an AU1-tag at the C-terminal end. The construct was inserted into the pFLAG-CMV3 vector downstream of an N-terminal preprotrypsin leader sequence followed by an N-terminal FLAG-tag. |
| DYKDDDDKAQVINTNSLSLLTQNNLNKSQSALGTAIERLSSGLRINSAKDDAAGQAIANRFTANIKGLTQASRNANDGISIAQTTEGALNEINNNLQRVRELAVQSANSTNSQSDLDSIQAEITQRLNEIDRVSGQTQFNGVKVLAQDNTLTIQVGANDGETIDIDLKQINSQTLGLDTLNVQISGGGGGILDSMGAEAAATTTENPLQKIDAALAQVDTLRSDLGAVQNRFNSAITNLGNTVNNLTSARSRIEDSDYATEVSNMSRAQILQQAGTSVLAQANQ__________GSEGKSSGSGSESKVTDSGSETGSSGSIPSCSFDGRIAFYRFCNLTQVPQVLNTTERLLLSFNYIRTVTASSFPFLEQLQLLELGSQYTPLTIDKEAFRNLPNLRILDLGSSKIYFLHPDAFQGLFHLFELRLYFCGLSDAVLKDGYFRNLKALTRLDLSKNQIRSLYLHPSFGKLNSLKSIDFSSNQIFLVCEHELEPLQGKTLSFFSLAANSLYSRVSVDWGKCMNPFRNMVLEILDVSGNGWTVDITGNFSNAISKSQAFSLILAHHIMGAGFGFHNIKDPDQNTFAGLARSSVRHLDLSHGFVFSLNSRVFETLKDLKVLNLAYNKINKIADEAFYGLDNLQVLNLSYNLLGELYSSNFYGLPKVAYIDLQKNHIAIIQDQTFKFLEKLQTLDLRDNALTTIHFIPSIPDIFLSGNKLVTLPKINLTANLIHLSENRLENLDILYFLLRVPHLQILILNQNRFSSCSGDQTPSENPSLEQLFLGENMLQLAWETELCWDVFEGLSHLQVLYLNHNYLNSLPPGVFSHLTALRGLSLNSNRLTVLSHNDLPANLEILDISRNQLLAPNPDVFVSLSVLDITHNKFICECELSTFINWLNHTNVTIAGPPADIYCVYPDSLSGVSLFSLSTEGCDEEEVLKSLKFSLFIVCTVTLTLFLMTILTVTKFRGFCFICYKTAQRLVFKDHPQGTEPDMYKYDAYLCFSSKDFTWVQNALLKHLDTQYSDQNRFNLCFEERDFVPGENRIANIQDAIWNSRKIVCLVSRHFLRDGWCLEAFSYAQGRCLSDLNSALIMVVVGSLSQYQLMKHQSIRGFVQKQQYLRWPEDLQDVGWFLHKLSQQILKKEKEKKKDNNIPLQTVATISDTYRYI#RS | |

Legend: restriction sites; linker sequence; tag sequence, sequence of HePy flagellin; aa mutation from HePy to SaTy
